# High-resolution sediment analysis reveals Middle Bronze Age byre-houses at the site of Oppeano (Verona province, NE Italy)

**DOI:** 10.1371/journal.pone.0272561

**Published:** 2022-08-31

**Authors:** Cristiano Nicosia, Federico Polisca, Christopher Miller, Bertrand Ligouis, Susan Mentzer, Claudia Mangani, Federica Gonzato

**Affiliations:** 1 Dipartimento di Geoscienze, Università di Padova, Padova, Italy; 2 Dipartimento dei Beni Culturali, Università di Padova, Padova, Italy; 3 Institute for Archaeological Sciences and Senckenberg Center for Human Evolution and Paleoenvironment, University of Tübingen, Tübingen, Germany; 4 SFF Centre for Early Sapiens Behaviour (SapienCE), University of Bergen, Bergen, Norway; 5 Museo Civico Archeologico Giovanni Rambotti, Desenzano del Garda, Italy; 6 Soprintendenza Archeologia, Belle Arti e Paesaggio per le province di Ravenna, Forlì-Cesena e Rimini, Ravenna, Italy; Universita degli Studi di Milano, ITALY

## Abstract

High-resolution sediment analysis allowed us to identify two Middle Bronze Age (MBA 1, 1650–1550 cal a BCE) byre-houses at the waterlogged site of Oppeano “4D”, south of Verona (Veneto region, NE Italy). The site lies in a low-lying valley incised by the Adige River in its LGM alluvial fan. In this fluvio-palustrine environment burial and taphonomic conditions were such that the archaeological record was extremely well preserved. The wooden elements making up basal parts of nine ‘huts’ were in fact exposed at Oppeano, and so were their internal accretion deposits. These featured finely laminated dung units deriving from the stalling of small herbivores, possibly ovicaprids, intercalated with repeated accumulations of wood ash. This was produced in large and multi-stratified hearths that were exposed within each hut. Organic petrology provided evidence of the production of wood tar inside one of the studied structures. At Oppeano 4D it was thus demonstrated that these structures were not just byres or stables, but spaces that housed humans together with animals at least during some periods of the year, hence byre-houses. The identification of byre-houses in a Middle Bronze Age settlement is key for the reconstruction of socio-economic aspects of Bronze Age economy and production systems.

## Introduction

Archaeological structures interpreted as byre-houses range from the Neolithic to the Middle Ages. Examples from the Bronze and Iron Age of northern Europe and Scandinavia are prevailing (see S1 Table and references therein). This is due to the numerous attestations of three-aisled longhouses (see [1] for The Netherlands; [2] for Denmark), that are systematically interpreted as a form of byre-house [3–6]. All archaeological structures interpreted as byre-houses are in fact longhouses (*sensu* [7]), featuring separated partitions for livestock and humans. To our knowledge, no comparable examples are present in the archaeological literature from southern Europe [8]. S1 Table provides an account of the type of data used to interpret archaeological structures as byre-houses across Europe (Neolithic to Middle Ages–for later periods see [9–13]). Most structures have been interpreted based on architectural characteristics, using particularly well-preserved contexts as references (e.g., Feddersen Wierde in Germany [14], Nørre Tranders in Denmark [15], Ezinge in The Netherlands [16]). Other data to interpret structures as byre-houses include the distribution and composition of macrofossils [17–21] and phosphate analysis [17, 22–29]. Various authors expressed doubts about the reliability of phosphate analysis as a proxy [30–32]. The literature review in S1 Table suggests that very few sites have *direct* evidence that sedimentary accretion within the structures was caused by concurrent animal gathering and human presence. These identifications as byre-houses, or even just as byres or stables, are therefore dubious [7]. Soil micromorphology provides a powerful tool to identify human activities and to characterize stabling deposits, yet none of the byre-houses in S1 Table were interpreted as such based on the study of sediments under the microscope. At Brecht-Zoegweg (Belgium), byre-houses were indeed studied with micromorphology, but the analysis focused only on the byre sector of the house, without considering the supposed domestic one [33]. At Aðalstræti 16 (Reykjavik, Iceland), instead, micromorphological evidence was only briefly discussed, and the authors suggested that a longhouse was occasionally used as a byre-dwelling due to the “presence of fragmented vegetal remains” [34].

**Fig 1:**
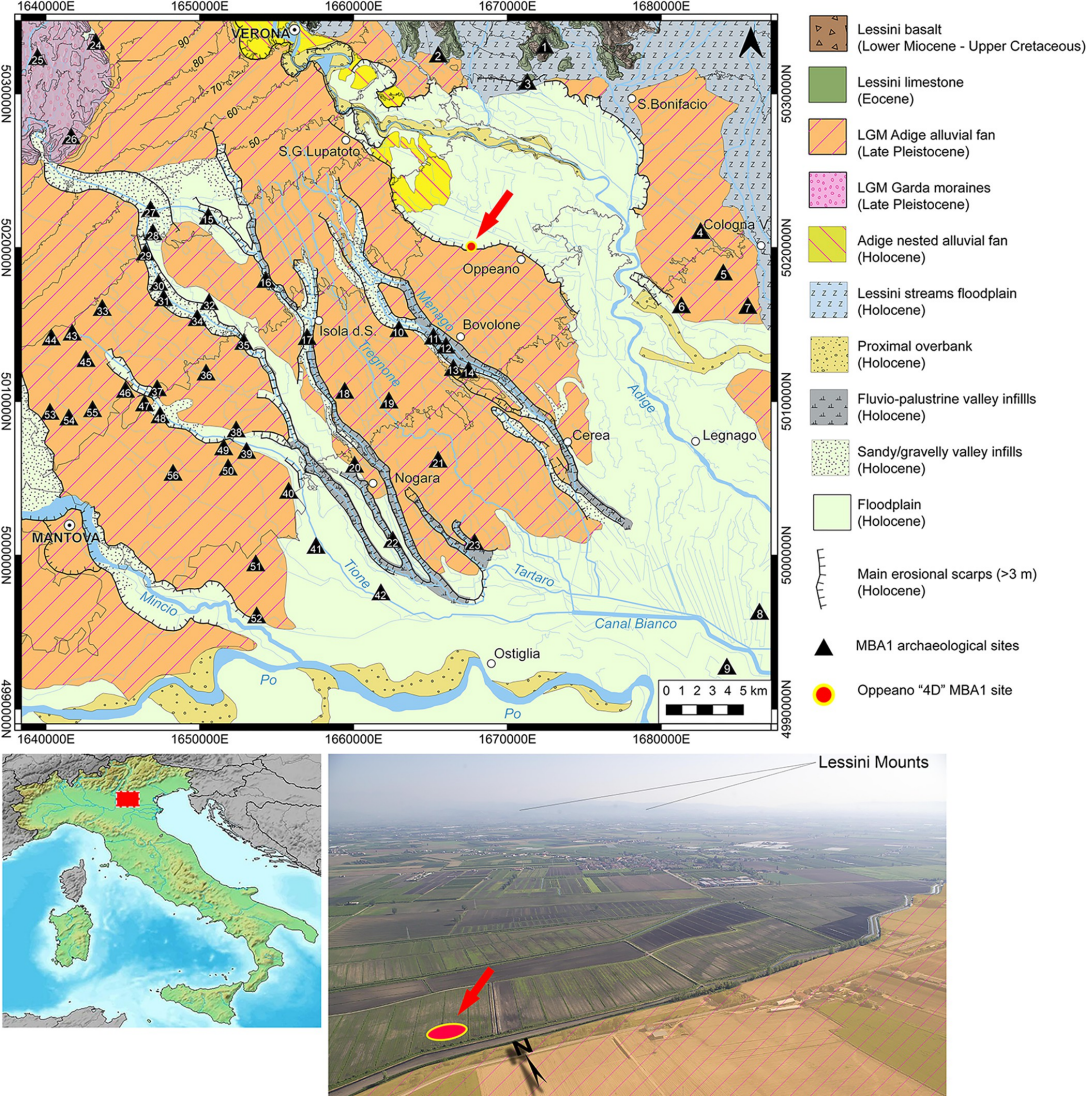
Geomorphological map showing the floodplain between the Lessini foothill and the present-day Po River. The map is based on: [35, 36], and Carta Geologica d’Italia 1:100.000 sheets n° 48, 49, 62, 63. The image of Italy in the lower left corner was obtained from Natural Earth (public domain—naturalearthdata.com). Fig 1 shows the position of the known EBA 2-MBA 1 and MBA 1 settlements (i.e., contemporaneous to Oppeano 4D; based on [37–40]): (1) Monte Castejon; (2) Corte Palù; (3) Monte Rocca; (4) Veronella–via Roversello 19; (5) Fondo Lora-Buratti; (6) Cimitero di Caselle; (7) Bernardine di Coriano; (8) Stanghelletti; (9) Marola; (10) Novarina; (11) Prà Longo di Tarmassia; (12) Le Gesiole; (13) Saccavezza; (14) Scolon di Saccavezza; (15) Isolalta; (16) Panzana; (17) Mulino Giarella; (18) Pellegrina; (19) Corte Olmi; (20) Montalto; (21) Barabò- via Barabò; (22) Il Mulino; (23) Maccacari–Quartieri Nord; (24) Monte Corno; (25) Torbiera Cascina; (26) La Palù di Sommacampagna; (27) Grezzanin; (28) La Muraiola di Povegliano; (29) Grezzano–Ortigara; (30) Prà grande; (31) I Camponi–Nogarole Rocca; (32) Corte Braette; (33) Mozzecane–Quarto del Tormine; (34) Corte Vivaro; (35) Castello di Trevenzuolo; (36) Corte Il Dazio; (37) Traversoni; (38) Demorta; (39) Susano; (40) Pomella; (41) Corte Carnarola; (42) Finilone Valle; (43) Castiglione Mantovano; (44) Roverbella; (45) Prestinari; (46) Canedole; (47) Corte Bertola; (48) Castelbelforte; (49) Cimitero di Bigarello; (50) Casazza; (51) Roncoferraro; (52) Casaletto; (53) Corte Pero; (54) Santa Lucia; (55) Fornasotto; (56) Corte Gandolfa.

In this paper, we use high-resolution sediment analysis to provide robust evidence of the concomitant presence of animals and humans within two protohistoric structures that, as such, are definable as “byre-houses” (see also Conclusions). The site of Oppeano 4D (the full name of the site is “Oppeano–Via Isolo–sito 4D”, hereafter referred to as “Oppeano 4D” Fig 1) represents one of the very few–if any–known examples of byre-house in southern Europe (see [34] about Italy). These structures contain large quantities of trampled herbivore dung revealed by soil micromorphology. Their interpretation as “byre-houses” and not just “byres” rests upon the identification, during excavation, of several features pointing to domestic activities within them. These encompass multi-stratified hearths, and various classes of archaeological materials such pottery, burnt bone, and finds related to textile production (see Discussion). Micromorphological analysis, on the other hand, revealed reliable indicators of food preparation and consumption, such as microscopic fragments of bone, burnt bone, charred cereal grains, fishbones etc.. Thanks to their exceptional preservation conditions, the byre-houses of Oppeano offer new possibilities to understand key socio-economic aspects of Middle Bronze Age communities and of human-animal relationships.

### Archaeological context

The Middle Bronze Age (hereafter MBA, 1650–1350 cal a BCE [41]) represents an important phase of demographic growth in northern Italy and–especially–in the Po Plain [39, 42]. During the MBA 1 (1650–1550 cal a BCE), settlements were mainly located in wetlands and low-lying zones, continuing settlement strategies that had developed during the Early Bronze Age (EBA, 2200–1650 cal a BCE) with the diffusion of pile-dwellings [40, 42, 43]. The position of the site of Oppeano 4D (Fig 1) makes no exception. The site lies within a wide incision that the Adige River cut into its late Pleistocene alluvial fan (i.e., a fluvio-glacial fan or sandur), an area bordered by steep scarps and subject to fluvio-palustrine sedimentation. In the Veneto region, between the MBA 1 and MBA 2 (the latter period dating to 1550–1450 cal a BCE) settlements spread on topographically higher portions of the plain [39], possibly in response to increased fluvial activity within the incised valleys and lower areas. This fluvial instability is attributed by some authors [44, 45] to wetter and colder conditions occurred during the Löbben glacial advance. Numerous sub-quadrangular embanked settlements were from this point on built on alluvial ridges and raised fluvial terraces. These villages, known as “Terramare”, had a precise internal organization with huts built directly on the ground or on elevated platforms, following the orientation of the perimetrical structures [46]. The northern Italian MBA communities based their economy mainly on agriculture ([47, 48] and references therein), herding [49], and metalworking, exploiting the available Alpine copper ores [50]. Extensive trade involving luxury objects (e.g., amber, imported pottery) with Central Europe, the Balkans, the eastern Mediterranean world, and the Baltic area are very well documented in the Terramare sites, as well as cultural contacts testified by the distribution of metal artifacts (e.g., Sauerbrunn-Boiu swords; see the discussion on this topic in [50, 51]) [42].

### 1.3 The site of Oppeano 4D

**Fig 2:**
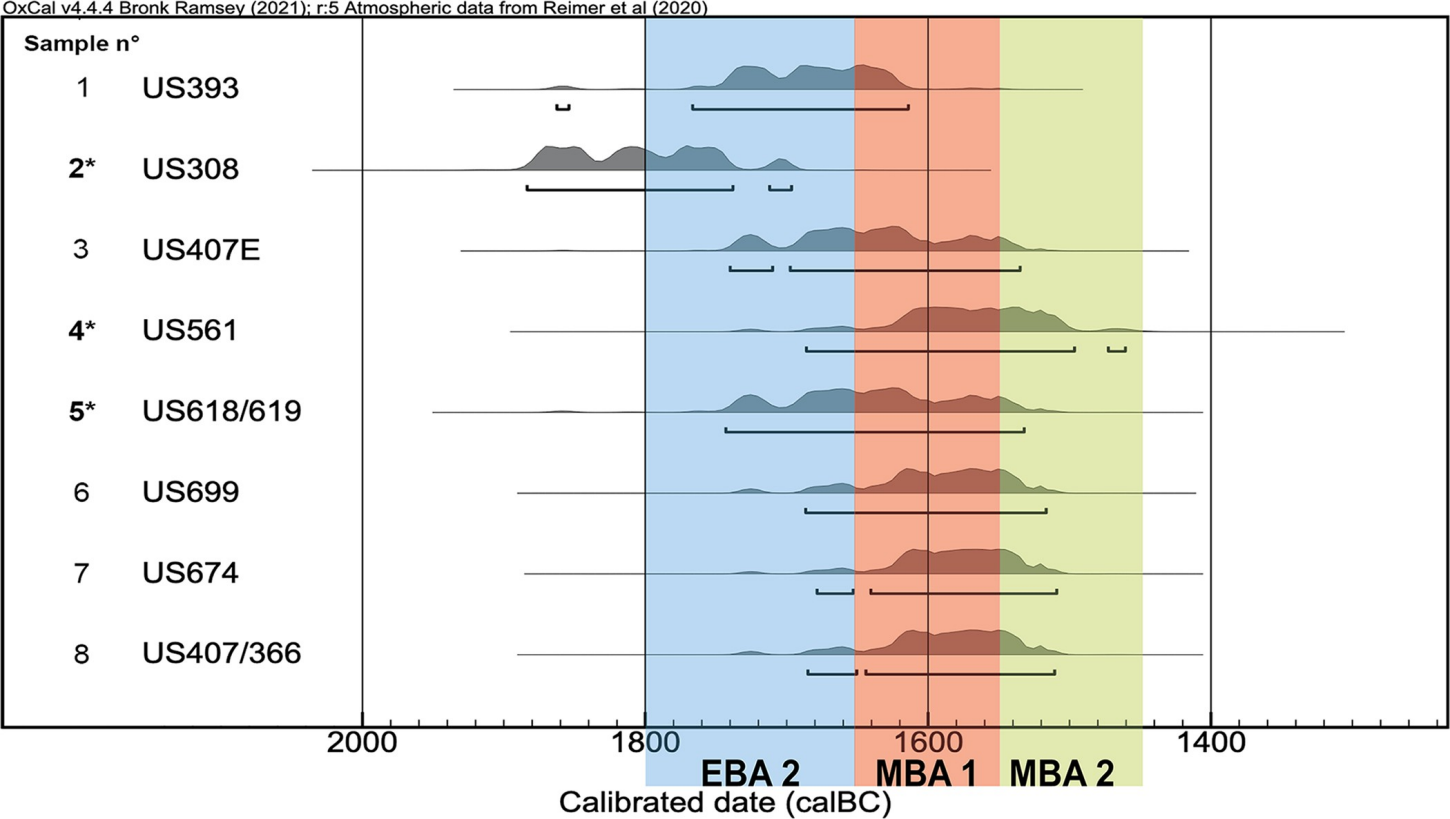
Radiocarbon dates related to the phase 2 of the site Oppeano 4D. Samples 2 and 5 = structure E; Sample 4 = structure F. The remaining samples come from other structures within phase 2 (see Fig 3): 1 (structure A);3, 8 (structure L); 6 (structure C); 7 (structure G). Bronze Age chronology based on [41]. See S2 Table for ^14^C dates and sample numbers.

Oppeano 4D is a waterlogged site excavated in 2014–2015 during rescue archaeological operations for the construction of a pipeline. Its geomorphic position (Fig 1), within a low-lying basin, is responsible for its permanently waterlogged status (the site had to be artificially drained to be excavated). The repeated arrival of distal overbank sediments from the Adige River to the North resulted in a rather deep burial (below 1–2 m from the present soil surface). The four phases that make up the site all fall within the MBA 1-2/3 (1650-1450/1350 BCE) time interval [52]. The second phase of the settlement–that is the one studied here–dates to the MBA 1 (1650–1550), as suggested by a preliminary assessment on pottery artifacts (Elisa Dalla Longa, per. comm. 2022) and ^14^C dates obtained from the structures discussed here and other coeval structures (Fig 2 –see also S2 Table). This phase was buried by a level of peat and gyttja formed as the result of a rapid shift towards wetter conditions, accompanied by a heightened groundwater level, in this part of the basin.

**Fig 3:**
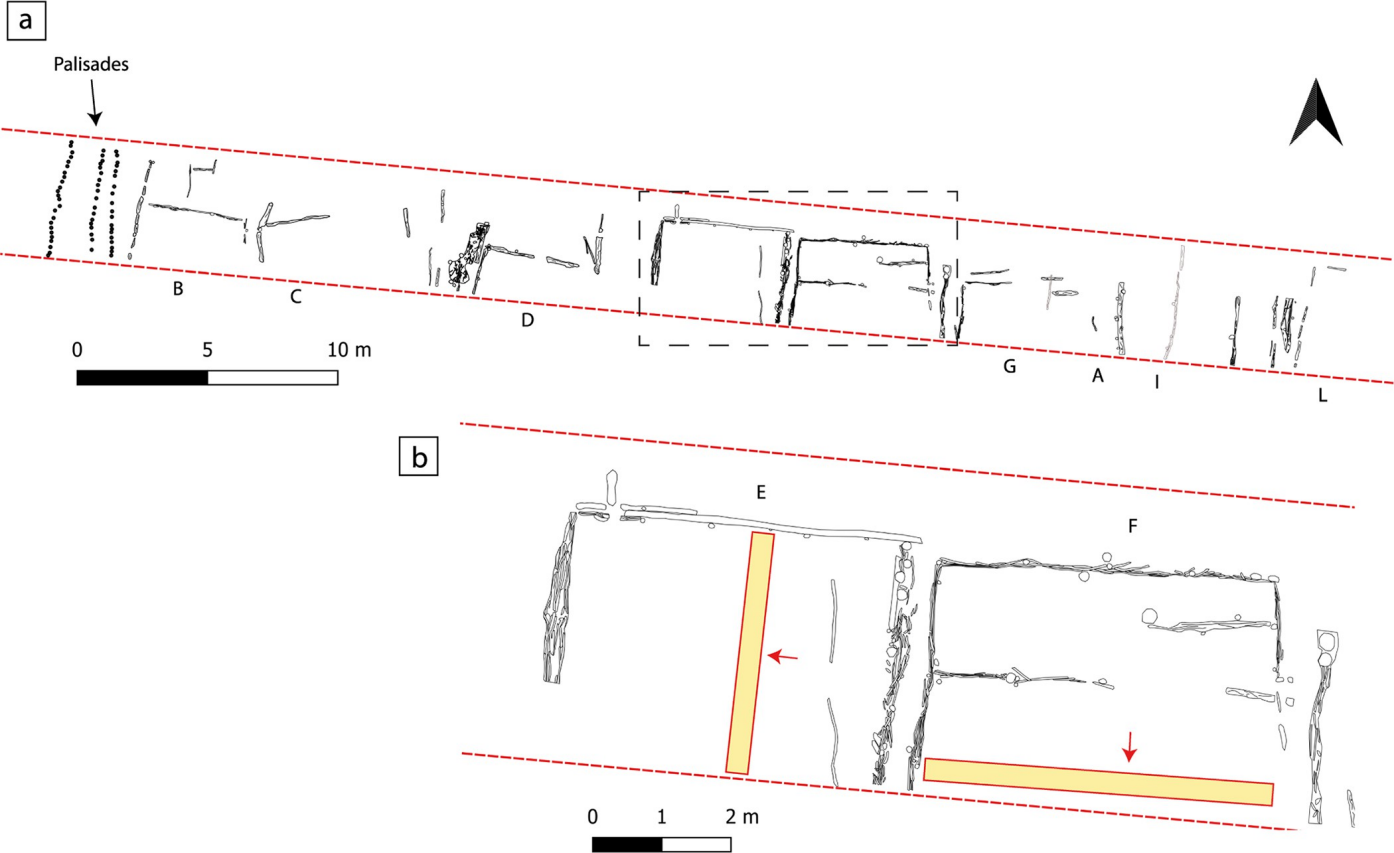
Excavation plan of the site Oppeano 4D and detail of structures E and F. (a) Plan with the nine structures excavated at Oppeano 4D and the palisades marking the eastern site limit. All structural elements belonging to the site’s phase 2 are displayed here. The dashed rectangle indicates the portion shown in (b), while the red dashed lines mark the excavation limits; (b) Detail of the excavation plan, showing structures E and F. The yellow rectangles indicate the location of the sampled baulks, the red arrows point at the side that was sampled. (Drawing: Michele Baldo).

**Fig 4:**
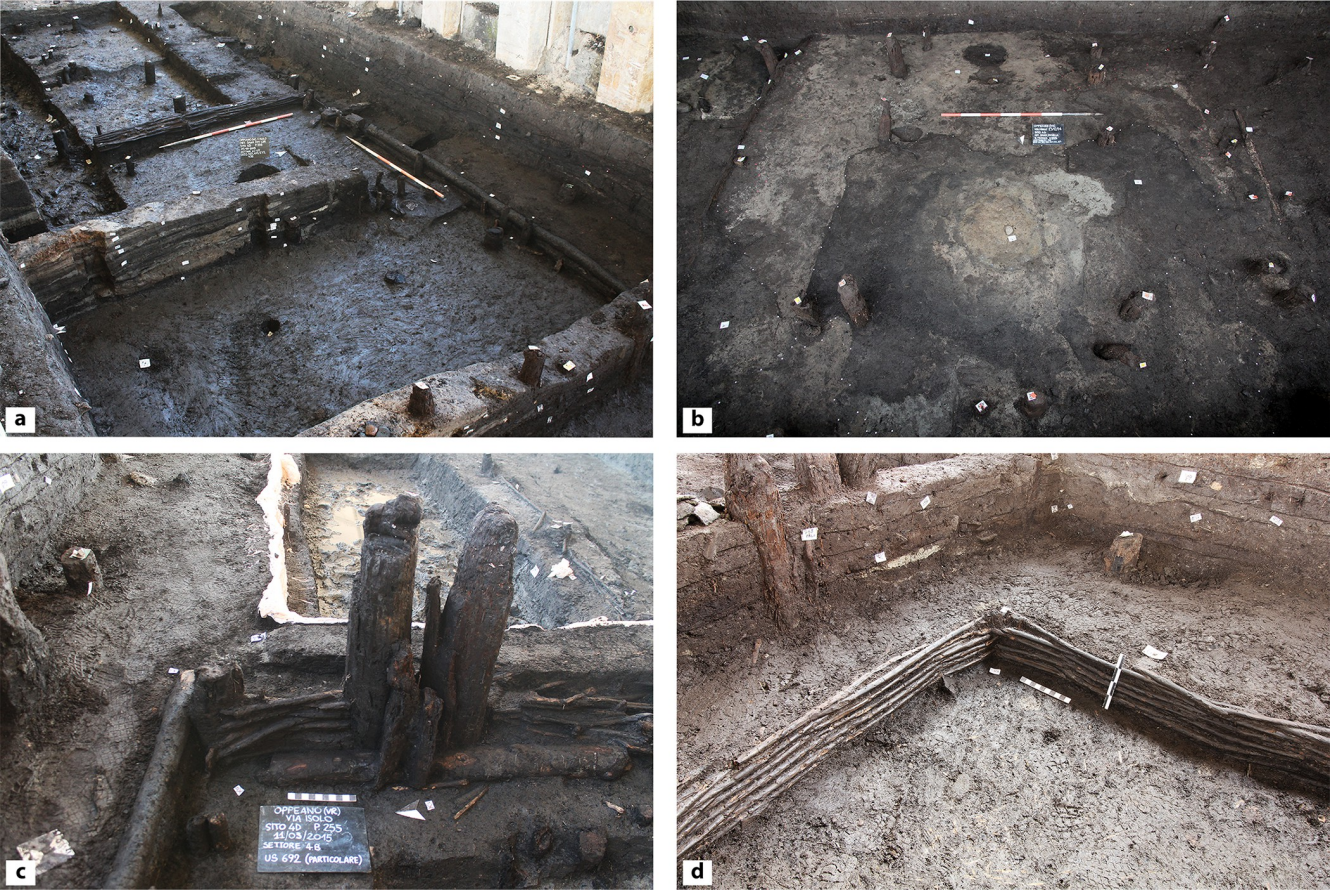
Field photographs. (a) general view of structure E, showing the walls built using different techniques and the internal stratification; (b) general view of structure A, characterized by two parallel rows of vertical posts, a central hearth, and a well-preserved sandy-clay floor; (c) detail of structure F walls, built using horizontally lying timber and branches woven around vertical posts (‘wattle and post’); (d) detail of a corner of structure F, showing walls built using the wattle and post technique.

The rapid burial and the onset of waterlogged conditions soon after the site abandonment allowed for the exceptional preservation of nine structures built originally on dry ground (Fig 3). The perimeter of these structures was clearly identifiable, as the lower portions of their walls were entirely preserved. These walls were built with branches woven around vertical posts (“wattle and posts” technique), with horizontally lying timber and planks, sometimes used in combination (Fig 4A and 4C). The structures were E-W aligned and divided by narrow alleys. The size of the excavation trench (70 m x 5 m) did not allow us to expose them in their entirety. The short side (northern) of the structures measured 5 m, while the long ones were exposed for maximum 4 m. Few structures dating to the MBA 1–2 and built on dry ground have been previously documented in the Verona province, so that it is not possible to make any hypothesis on the original size of our buildings. The only comparable site–Muraiola di Povegliano (site no. 28 in Fig 1)–featured two rectangular huts smaller than the Oppeano ones, as their internal surface was ca. 15 m^2^ (5,40 m x 3,10 m) [53]. At Oppeano, stratigraphic relationships indicate that eight of the huts (B-L, see Fig 3) were contemporaneous. They were in fact built directly on an organic-rich backfill put in place prior to the construction of the village. A ninth structure (A), instead, was built over the area formerly occupied by structures G and I, after they were abandoned and sealed by a ground-levelling layer (Fig 3). The resolution of radiocarbon dates and ceramic typo-chronology largely confirm the picture provided by stratigraphy, yet at the same time does not allow for the construction of a tighter chronological frame (see S2 Table).

**Fig 5:**
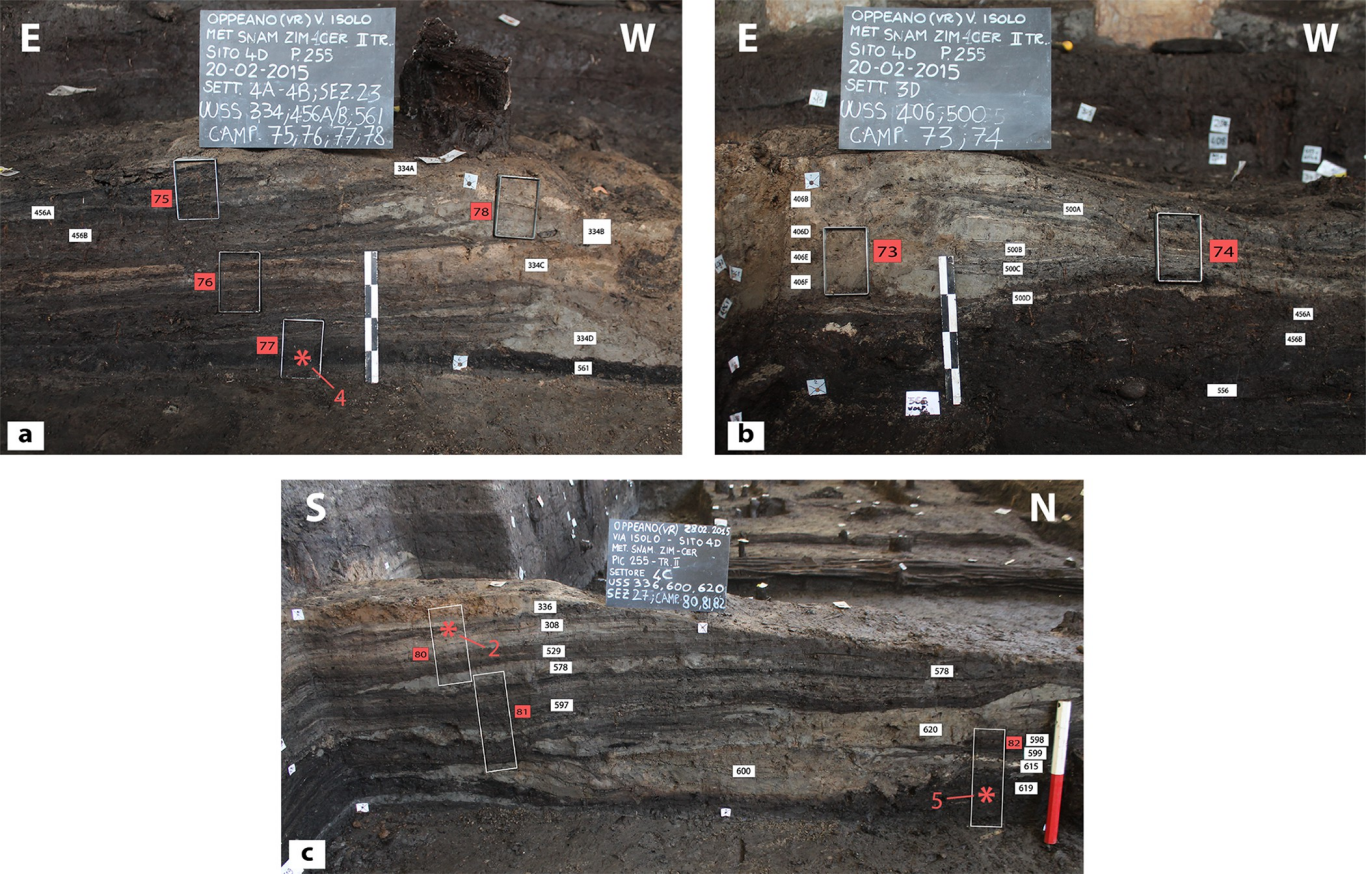
Profiles showing the internal stratification of structures E and F. Red rectangles mark the samples’ positions; white rectangles refer the stratigraphic units (US) identified in the field; asterisks indicate the sampling location for the ^14^C dates (see Fig 2 and S2 Table). (a) Internal stratification of structure F (samples 75–78; sample 79 is not shown, it was collected immediately west of sample 77); (b) same profile as (a) but showing a portion located further E; (c) Internal stratification of structure E (monoliths 80–82).

Finely laminated deposits resulting from daily activities accumulated on floors inside each structure. Such floors were built with local organic silts or with sandy clays quarried from the rubified Alfisols that blanketed the nearby Adige alluvial fan (Fig 4B). Thanks to the peculiar depositional history of the site, the internal deposits suffered no bioturbation, and had all their organic components (i.e., vegetal fragments, dung, seeds) preserved. Numerous hearth preparations intercalated with organic-rich deposits and featuring multiple phases of renovation and replastering (Fig 5) were exposed. This paper focuses on the high-resolution study of the internal accretions in two of the nine excavated structures, namely structures E and F (Figs 3–5).

## Material and methods

A 50–100 cm wide stratigraphic baulk crossing the internal part of each structure was left unexcavated in order to be sampled. Kubiena boxes were employed to collect soil micromorphology samples (Figs 3 and 5). Bulk samples were collected from the main units discernible in the field in close conjunction with micromorphology blocks. These were also subsampled prior to embedding with resin. Samples were air dried (i.e., no acetone replacement) and manufactured according to the methods of Murphy [54]. Their description followed the terminology of Stoops [55]. Soil Microfabric Types (hereafter, SMT) were employed to describe the sequences, as these were finely laminated and featured recurrent sub-units (i.e., further subdivisions of the field stratigraphic units based on micromorphology). Each SMT corresponds to a set of micromorphological characteristics which can occur within different thin sections or different sub-units of the same thin section [56, 57]. They are labelled with progressive numbers, and variations within the same SMT are indicated by a small letter (e.g., SMT 1, 1a, 1b). Micro XRF mapping was performed on uncovered thin sections at the Institute for Archaeological Sciences of the University of Tübingen [58]. Organic petrology was performed on one sample to differentiate between charred and humified blackish plant residues [59]. These analyses were conducted on the polished block resulting from thin section production (sample OPP77). The analysis was performed in reflected light [60]. The description and classification of organic micro-components (macerals) is based on the nomenclature of macerals in brown coal and coal [60, 61]. AMS ^14^C dating was performed at IFIN-HH Nuclear Physics Department in Bucharest (Romania) and at the Centre for Isotope Research (CIO) of the University of Groningen (The Netherlands) (Fig 2 –see S2 Table).

## Results

**Fig 6:**
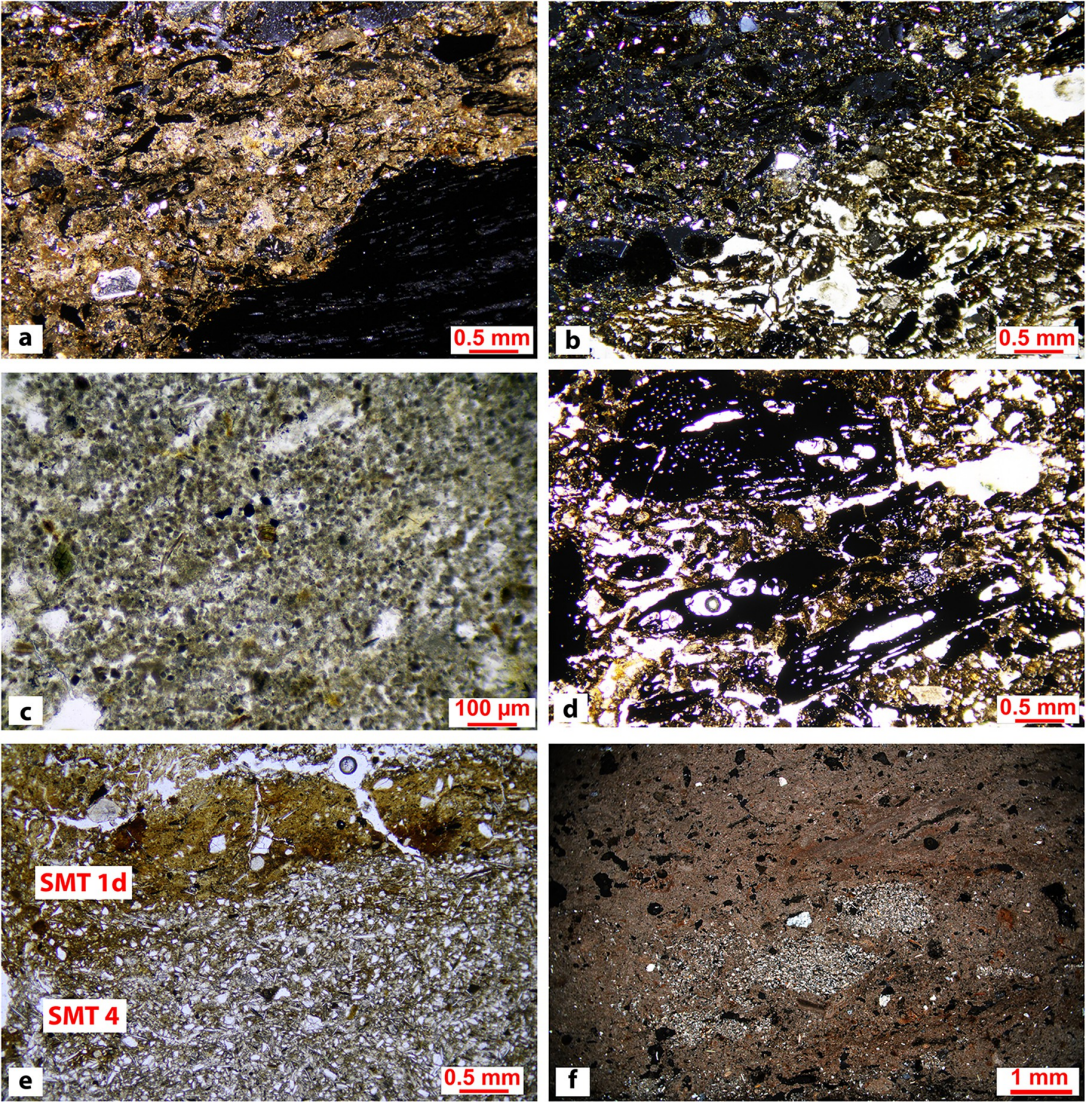
Microphotographs of SMT 1, its sub-types, and SMT 4. (a) SMT 1: ashes mixed with charcoal and charred vegetal matter (XPL). Note the compaction of the material and the alignment of coarser components due to trampling; (b) SMT 1a: compacted ash with charcoal and herbivore dung. The birefringent particles are predominantly ashes, while the optically isotropic fragments are plant material related to herbivore dung (left half: XPL; right half: PPL); (c) SMT 1b: pure wood ash sub-unit (PPL); (d): SMT 1c: coarse charcoal fragments in an ashy matrix. Note the strong alignment of the coarser components due to trampling (PPL); (e): on top, SMT 1d: phosphatic crust, including quartz grains and mica. At the base, SMT 4: hearth preparation, showing a very dense and compact groundmass, composed of sandy silt (mainly quartz grains, mica). Note the abrupt limit between the two sub-units (PPL); (f): SMT 1e: aggregates from dismantled hearth preparations (reworked SMT 4 fragments) included in a pure ash groundmass similar to SMT 1b (XPL).

**Fig 7:**
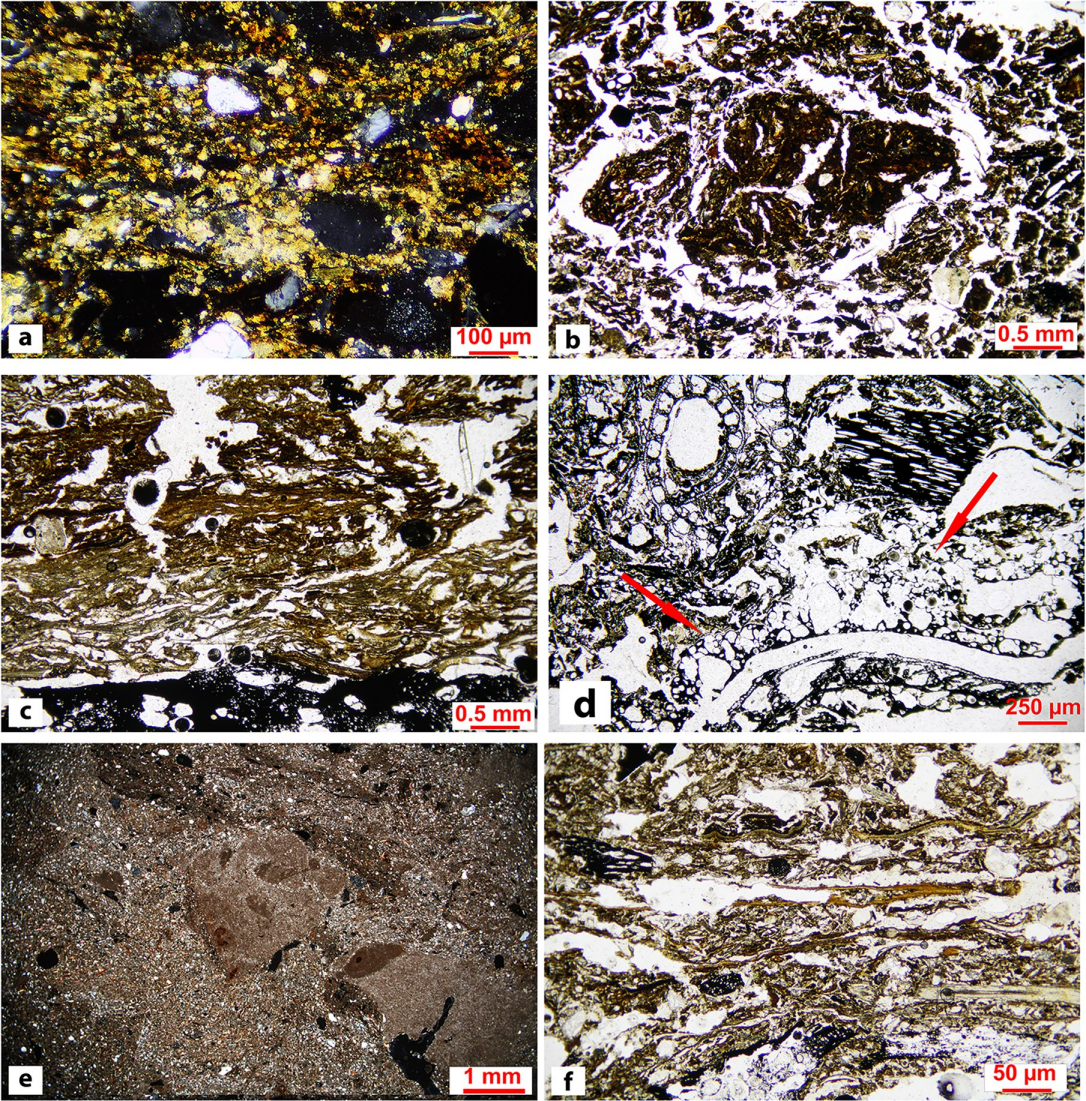
Microphotographs of SMT 2, its sub-types, SMT 3, SMT 4a, and SMT 5. (a) SMT 2: wood ash at the center of the image (brighter area) sandwiched between compacted and aligned herbivore dung. Note the presence of fecal spherulites in dung (XPL); (b) SMT 2a: non-compacted herbivore dung. Note the fragmented small herbivore coprolite, probably related to goat/sheep (PPL); (c) SMT 2b: compacted and aligned (i.e., trampled) herbivore dung on top of a charcoal fragment. (PPL); (d) SMT 3: Wood tar particle (red arrows) surrounded by charcoal and charred vegetal fragments. Note the numerous vesicles in the wood tar (PPL); (e) SMT 4a: heterogeneous hearth preparation made of silty sand/sandy silt sediments (cf. Fig 6E), including fine-grained calcareous aggregates (XPL); (f) SMT 5: gyttja floor. Note the massive microstructure and the abundance of vegetal fragments mixed with charcoal in a silty groundmass (PPL).

In total, five main SMT were identified, with 13 sub-types (see Figs 6 and 7 and Table 1 for a summary of the main characteristics of each SMT), across the 175 sub-units that were identified in thin section (see S1 Appendix).

**Table 1:** Table reporting the main characteristics (microstructure, diagnostic inclusions, orientation of the coarse components) and interpretation of each SMT and subtype.

SMT	Main characteristics			Interpretation
	Microstructure	Diagnostic inclusions	Orientation coarse components	
**1**	Massive	Wood ash	Parallel and horizontal	Hearth rake-out and trampling. Wood as main combustible
		Charcoal		
		Vitrified phytoliths		
		Chaff		
		Bone fragments		
**1a**	Massive	Same as 1	Parallel and horizontal	Same as 1 with presence of herbivores close to the structure
		Herbivore dung		
		Mineral trample		
**1b**	Massive	Wood ash	Parallel and horizontal	Same as 1, but in more oxidizing burning conditions
		Fine charcoal		
		Articulated phytoliths		
**1c**	Massive	Charcoal	Parallel and horizontal	Same as 1, but in more reducing burning conditions
		Wood ash		
**1d**	Massive	Fecal spherulites	/	Same as 1a
		Bone fragments		
**1e**	Massive	Same as 1b	Parallel and horizontal	Same as 1, but also destruction and reconstruction of hearths
		Aggregates of SMT 4		
**2**	Microlaminated undulating	Herbivore dung	Parallel and horizontal	In-situ stabling activities.
		Wood ash		Trampled
		Charcoal		
		Bone fragments		
		Mineral trample		
**2a**	Subangular blocky	Herbivore dung	Random	In-situ stabling activities.
				Non trampled
		Complete ovicaprid		
		excrement pellets		
**2b**	Microlaminated undulating	Herbivore dung	Parallel and horizontal	In-situ stabling activities.
				Trampled
**3**	Massive	Wood tar	Random	Wood tar production
		Charcoal		
		Plant residues		
**4**	Massive	Quartz	Mica shows preferential orientations	Hearth preparation
		Mica		
		Micritic aggregates		
**4a**	Massive	Same as 4	Same as 4	Heterogeneous hearth preparation
		Dismantled hearth preparations		
		Pottery fragments		
		Overbank calcareous clay-silt aggregates		
**5**	Massive	Vegetal tissue and fragments	Random	Organic-rich floor or ground raising layer
		Quartz		
		Mica		
		Charcoal		

### SMT 1: Ash-dominated microfabric types

Ashes dominated by prismatic microcrystalline carbonate pseudomorphs after calcium oxalates [62] and containing wood charcoal indicate that shrubs and trees were used as combustibles. Grasses and chaff were also burnt, as indicated by the presence of elongated charred vegetal tissue fragments, and of blackened or vitrified phytoliths. Notwithstanding the ubiquitous presence of herbivore dung in the studied sequences, no trace of its use as combustible was observed in any of the thin sections, as not a single blackened spherulite was observed [63]. In SMT 1 ashes are predominantly compacted and coarser components (charcoal, vegetal tissue and organic fragments, bone, shell) are horizontally aligned, indicating trampling [64].

In SMT 1a unburnt dung is present alongside wood ash–still the dominant component—and charcoal. The dung fragments and scattered spherulites that characterize SMT 1a are most likely brought in by trampling and, as such, mixed in within the ashes. The input of “trample” [65] is also indicated by the presence of thin, elongated, minerogenic aggregates composed of micaceous sandy loam-textured sediments. Their lithology and granulometry reflects those of the local alluvial substrate (see SMT 4).

SMT 1b was assigned to sub-units consisting of almost exclusively wood ash, with minor quantities of finely comminuted charcoal, a characteristic that gives them a brighter colour in both PPL and XPL. This SMT can have a vesicular microstructure or be massive and is interpreted as deriving from more prolonged burning episodes or more fully oxidizing conditions with respect to other ash-dominated SMTs [66]. Yellowish iron-phosphatic mottles are common post-depositional features in SMT 1b, possibly derived from weathering of ash [67]. Still, they are rather widespread throughout the sequence, and often form in and around larger charcoal fragments, as evidenced by μXRF analysis.

SMT 1c is characterized by a close porphyric related distribution pattern in which the coarser elements are wood charcoal fragments, and the fine matrix between them is wood ash. These almost monogranular sub-units in which charcoal fragments are horizontally aligned derive from hearth rake-out and trampling [64, 68].

SMT 1d, observed only in thin section OPP 80–1, is characterized by phosphatic crusts in a calcareous silty clay groundmass that also includes fecal spherulites and snapped bones. Similarly to SMT 1b, these phosphatic features might derive from ash weathering [67].

SMT 1e is a rather heterogeneous SMT and was only observed in thin section OPP 80–2. In it the groundmass is similar to the “clean” ashes of SMT 1b, but with several rounded fragments of hearth preparations (reworked SMT 4 fragments), dung fragments, and aggregates of fused phytoliths. This can be interpreted as the result of the destruction and reconstruction of hearths.

### SMT 2: Herbivore dung-dominated microfabric types

In this SMT herbivore dung predominates over ash or constitutes the sole component. Based solely on optical properties, we can only state that the dung in the studied structures is of herbivore origin [69]. Input of faeces from omnivore-carnivores (humans, pigs, dogs) is absent in the studied thin sections. It seems reasonable to suggest that the observed herbivore dung is from small herbivores such as sheep or goat, based on: (a) the morphology of some whole dung pellets observed in thin sections OPP 77 (sub-unit 8) and; (b) the lack of indicators of surface puddling, poaching, and urine-related slacking, that is commonly observed in stables where cattle is penned [64]. For a more precise determination of the producer of the observed dung further analyses (i.e., parasites, DNA, fecal sterols and stanols, and bile acids) will be necessary.

Dung is preserved mostly as laminated and compacted levels of vegetal tissue and organic residues, phytoliths, and fecal spherulites, ranging from a few microns to 1–2 centimetres in thickness. Ash, bone fragments, mineral trample, and small ceramic fragments are intermixed within the thicker dung-dominated sub-units, as a result of repeated trampling within the structures. SMT 2 corresponds to this latter type of material. In it the horizontal parallel orientation of coarser components and the laminated or undulating laminated aspect of dung points to repeated trampling and compaction by livestock [70–72].

SMT 2a was assigned to sub-units in which dung lacked the compression and lamination features, resulting in chaotically distributed, often complete, ovicaprid excrement pellets.

SMT 2b identifies pure dung accumulations, which are normally extremely thin (from ca. 50 μm to 2–3 mm). These are normally sandwiched between ash-rich sub-units, and possibly testify very brief episodes of circulating livestock within the structures prior to ash spreading and to the “reset” of the dwelling surface.

**Fig 8:**
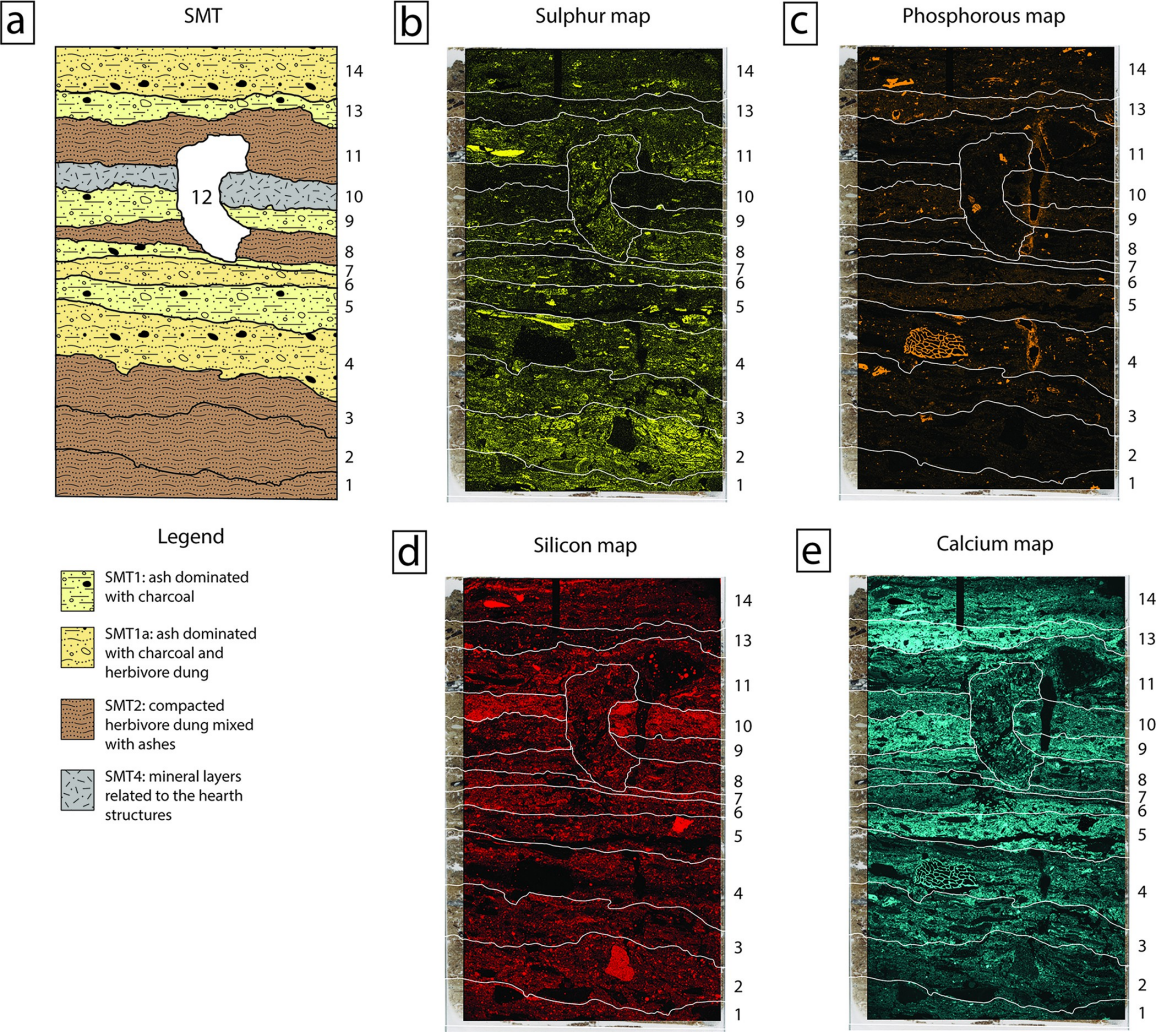
Interpretation of thin section OPP 76 (structure F) and related μXRF map. (a) Interpretation of thin section OPP 76, with a SMT assigned to each identified sub-unit. Note the alternating sequence of SMT 2 (dung mixed with ashes) and SMT 1 (ash mixed with charcoal)/SMT 1a (ash mixed with herbivore dung and charcoal). Non-interpreted version of thin section OPP 76 is available in S1 Appendix; (b-e) μXRF maps showing the abundance of specific elements superimposed on the PPL scan of thin section OPP 76. In (b), note the correspondence between high S signal and SMT 2 sub-units and–in minor amount–SMT 1a sub-units that still contain fragments of herbivore dung. In (c) and (e), note that the highest concentrations of P and Ca are visible in SMT 1 sub-units (n° 5, 9, 13), while SMT 2 sub-units show very low P values. In SMT 2, the coarse elements with high P signal are bone fragments.

From a chemical standpoint, SMT 2 sub-units were characterized by a dominant signal of sulphur in the μXRF maps, while phosphorus, that is traditionally interpreted as a fecal signal (see [66] and references therein), usually showed very low values (see Fig 8).

### SMT 3: Sub-units with dominance of charred and/or humified plant residue

SMT 3 corresponds to randomly oriented charred vegetal fragments and plant residues in a groundmass which included a minor amount of mineral (quartz, mica, and micrite) and anthropogenic components (ash and pottery). Considering the limit of micromorphology in distinguishing between charred and humified plant tissues, organic petrology allowed for a better characterization of SMT 3 [59]. This SMT is composed of numerous fusinite tissues (charcoals and burned tissues) showing *in situ* tar and detrital tar particles no longer attached to the tissues from which they were generated. The tar particles exhibit generally a low size heterogeneity (up to 700 μm), even if fine granular tar was also present. Tar fragments showed numerous vesicles of variable shape and size and they often have fine porosity. High reflecting tar particles (i.e., with reflectance higher than 5.00%Ro) showed an incipient anisotropy.

**Fig 9:**
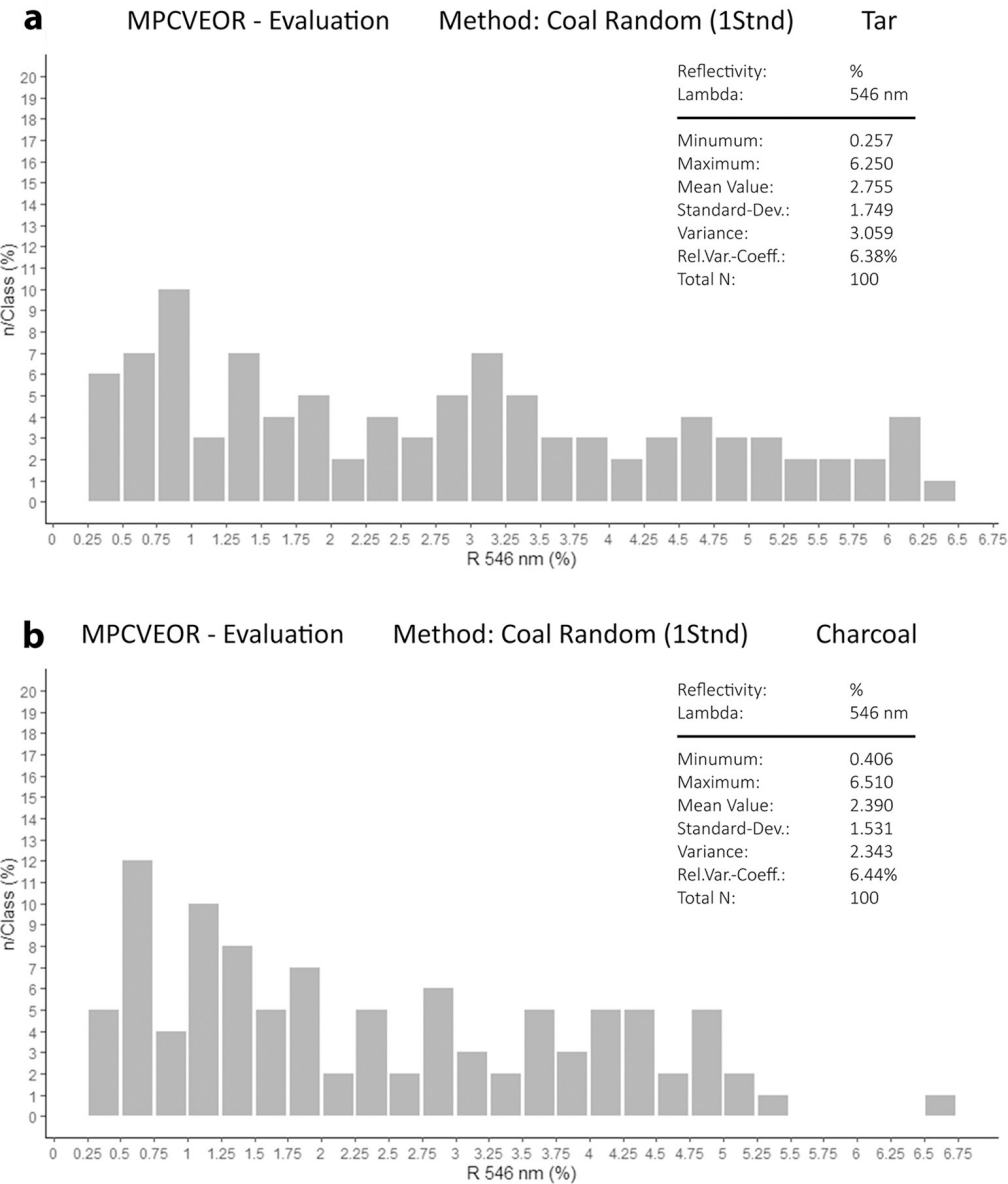
Reflectance histograms. (a) Values measured on tar particles, and (b) on charcoal/burnt tissues from sub-unit n° 7 of thin section OPP 77. Charcoal measures were taken both on specimen associated with tar and not (see raw data and R code in S1 Dataset and S1 File respectively).

In Fig 9A, the tar reflectance histogram shows a continuous and almost regular distribution of reflectance values ranging from 0.25 to 6.25%Ro that follows the one of the associated fusinite tissues (from 0.41 to 6.51%Ro, see Fig 9B). All the categories of charcoal reflectance are represented, from very low to very high (semifusinite, fusinite, pyrofusinite), although the low reflecting fusinite interval (0.50 to 1.50%Ro) shows a somewhat higher frequency. Overall, the reflectance indicates formation temperatures approximating from 235°C to 820°C [73], while the values located outside the histogram suggest a temperature of 950°C. The unusually broad histogram of reflectance showing a continuous distribution value–and therefore of temperature–can be interpreted as the result of careful heat control obtained by limiting the oxygen supply. This was aimed at obtaining a restricted burning and it probably indicates the will to produce wood tar. In addition to tar, SMT 3 contained unburned vegetal detritus, poorly preserved telohuminite tissues and birch-derived suberinite tissues. It also contains pyrite mostly occurring in tissues as single microcrystals and framboïds (up to 40 μm in size). These most likely derive from fast oxidation occurring as groundwater was artificially lowered to perform the excavation.

### SMT 4: Minerogenic hearth preparations

Hearth preparations are mainly composed of silty sand/sandy silt dominated by quartz, mica, and very fine to fine sand-sized micritic aggregates occurring in a calcareous micromass. Other geogenic inclusions, such as feldspars, chert, and rock fragments, are sporadic. Overall, the lithological composition of SMT 4 sub-units is perfectly in line with that of the sediments of the Adige alluvial plain [74]. Very rarely centimetric anthropogenic aggregates of ashes related to SMT 1 and SMT1b were found dispersed in the SMT 4 sub-units.

The boundaries of the hearth preparations are usually abrupt and undulated, showing plastic deformations caused by downward pressures from the units above, or water-escape features from the materials below. The latter consisted in flames of silty sediments protruding in the SMT4 sub-unit from below that are indicative of high-water content. According to Karkanas [75], these features are the product of the squeezing of water‐enriched domains along planes of weakness. Plastic deformations were also visible in the perpendicular orientation of mica with respect to pores, and in the preferential parallel orientation around coarse inclusions, showing examples of rotational core grain structures [75]. These characteristics suggest that patches of sandy silt were kneaded with water to obtain a plastic mixture that was then put in place on a humid surface.

The subtype SMT 4a was assigned to minerogenic sub-units characterized by a greater compositional heterogeneity. They include fragments of dismantled hearth preparations, pottery fragments, and overbank calcareous clay-silt aggregates. The reuse of construction materials to renew hearth has been documented also in other coeval sites in northern Italy (i.e., at the Lavagnone pile dwelling) [76].

### SMT 5: Organic silt/gyttja floors

This SMT was observed only in thin section OPP 82–1 (Fig 5C). It features organic silt with vegetal tissue fragments and a compact (i.e., massive) microstructure. These sediments were readily available in the immediate surroundings of the site. Floors or ground-raising layers with a similar composition were observed in several structures during excavation. They often contained abundant twigs and bark, possibly with the function of keeping floors dry [77].

## Discussion

**Fig 10:**
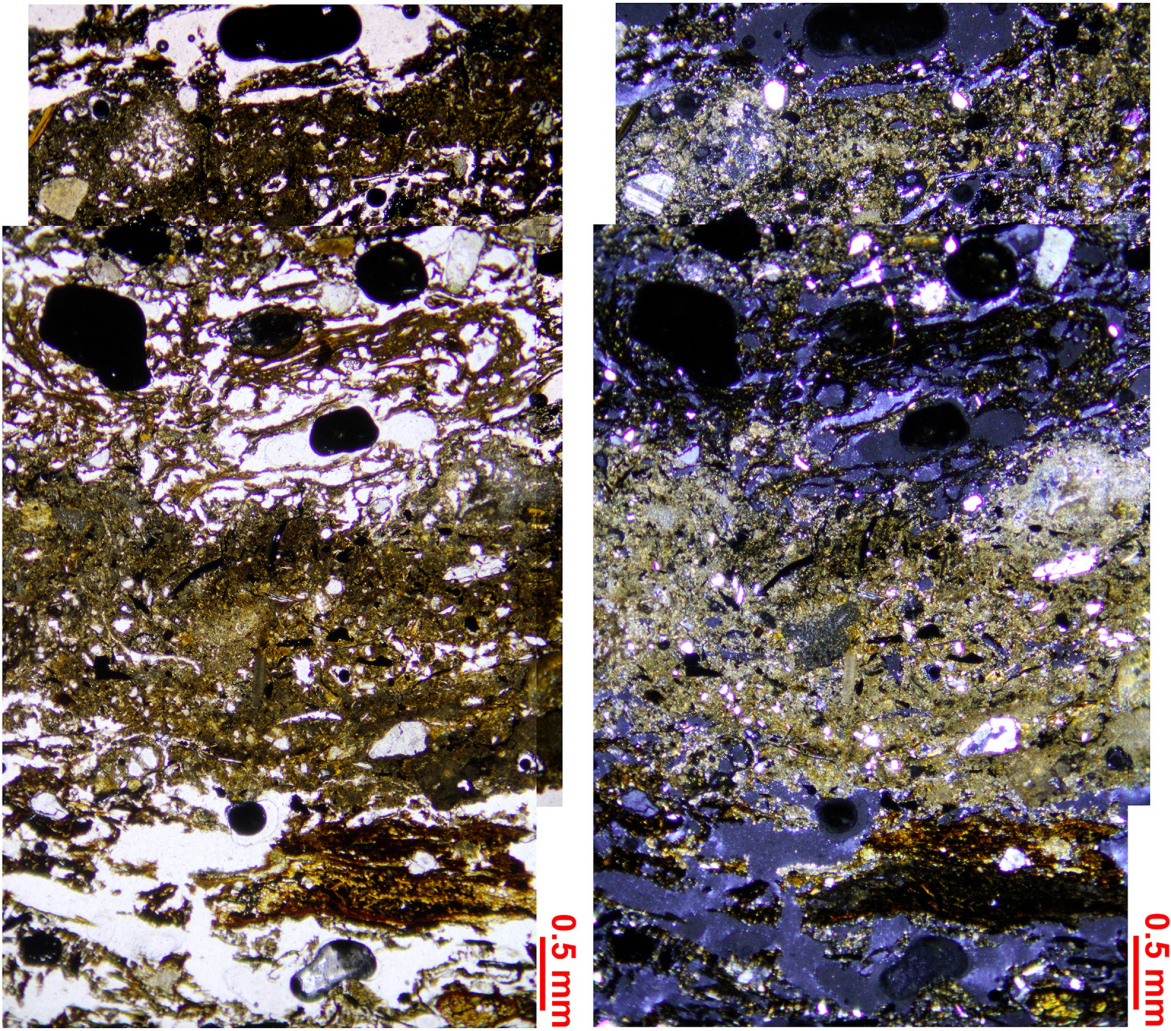
Typical alternating sequence of ash (SMT 1, SMT 1b) and trampled herbivore dung (SMT 2b), recurring several times in both studied structures. Structure E, thin section OPP 82–3. PPL (left) and XPL (right).

**Fig 11:**
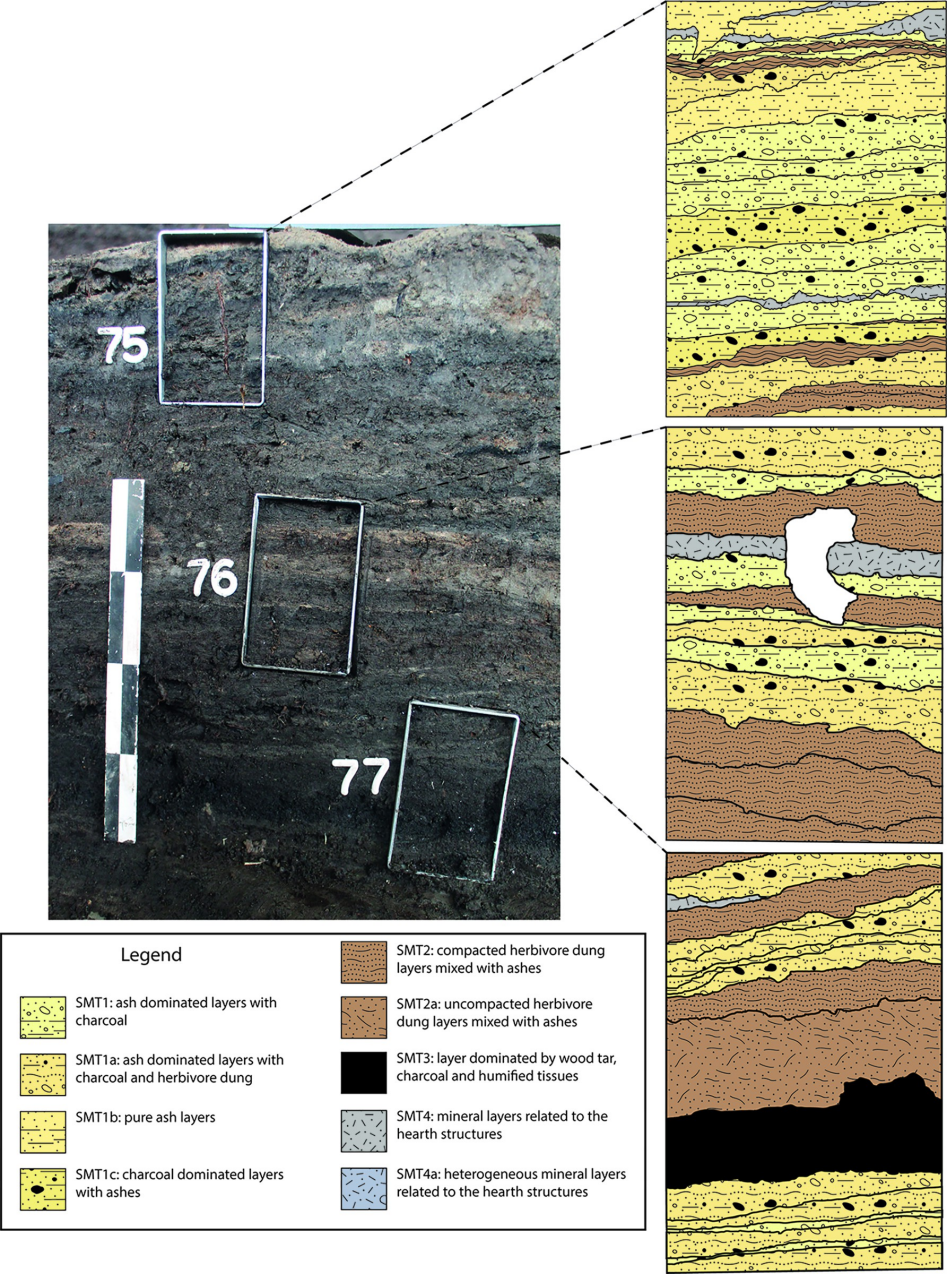
Sampling location of OPP 75–77 and interpretation of the vertical accretion of structure F. Left: detail of Fig 5A, showing the position of samples OPP 75, OPP 76, and OPP 77. Right: interpretation of the thin sections, with a SMT assigned to each sub-unit.

**Fig 12:**
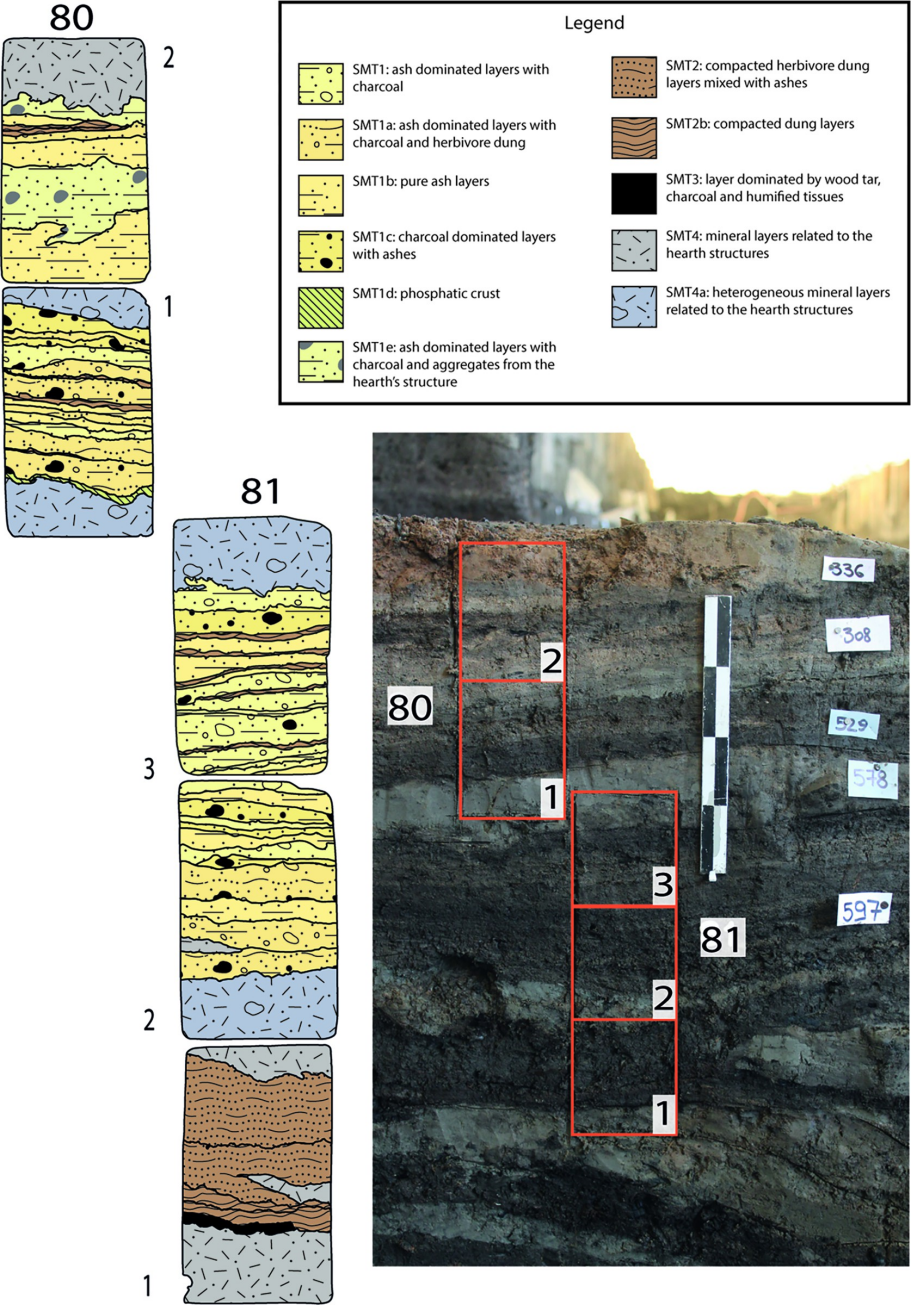
Sampling location of OPP 80–81 and interpretation of the vertical accretion of structure E. Right: detail of Fig 5C, showing the position of monoliths OPP 80 and 81. Two thin sections were obtained from OPP 80 (80–1, 80–2), while monolith OPP 81 provided three thin sections (81–1, 81–2, 81–3). Left: interpretation of the thin sections, with a SMT assigned to each sub-unit.

**Fig 13:**
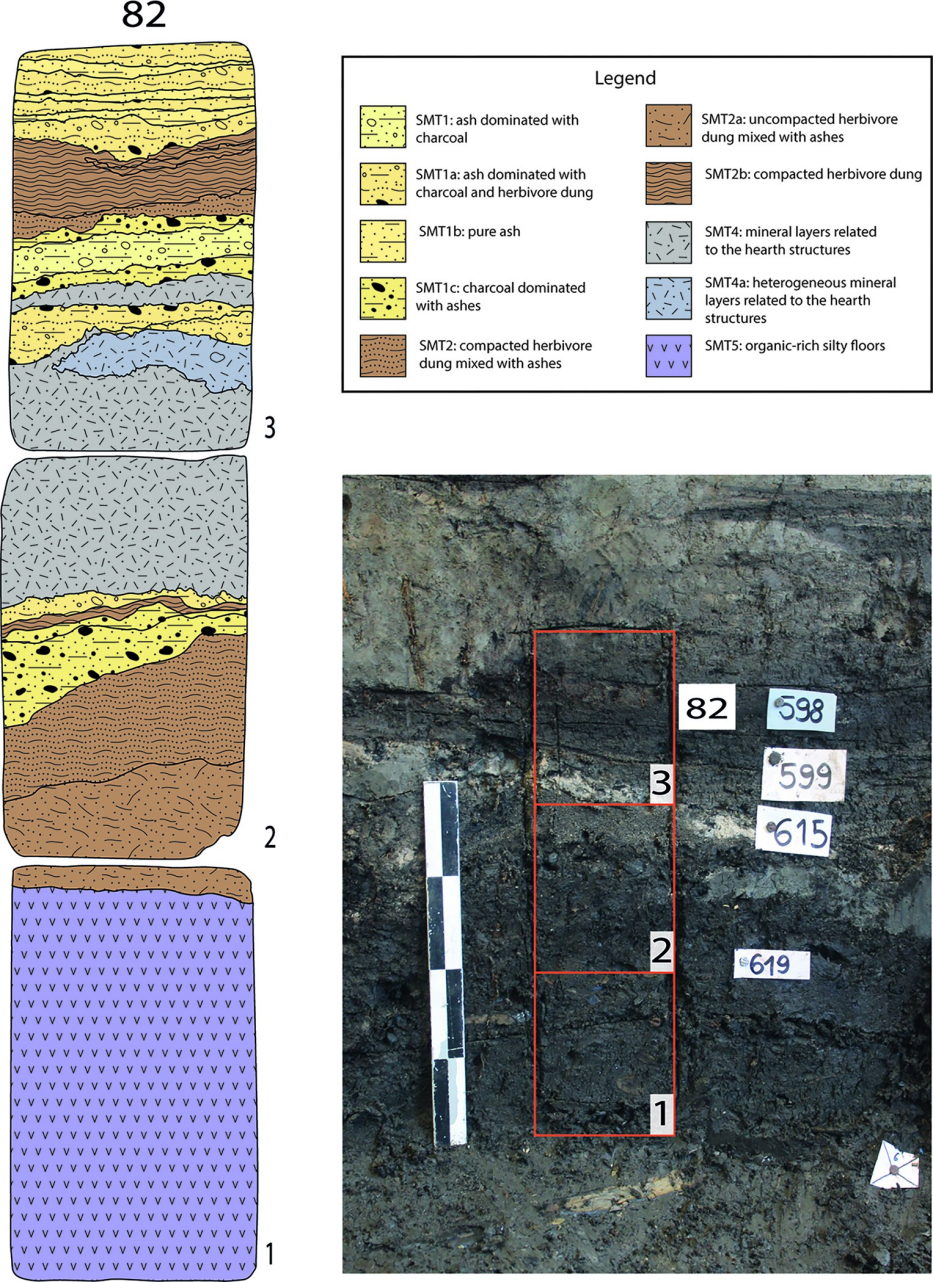
Sampling location of OPP 80–81 and interpretation of the vertical accretion of structure E. Right: detail of Fig 5C, showing the position of monolith OPP 82. Three thin sections were obtained from OPP 80 (82–1, 82–2, 82–3). Left: interpretation of the thin sections, with a SMT assigned to each sub-unit.

High-resolution sediment analysis demonstrated that the internal accretion in the two studied structures derives mainly from the repeated accumulation of herbivore dung and of wood ash and rake-out from hearths (Figs 10–13). This repeated alternation was observed on millimetric to centimetric units that in the field were laterally continuous, and therefore not confined to localized portions of the deposit. The excavation of the herbivore dung and intercalated wood ash level unearthed several artefacts indicating that in the studied structures domestic activities took place alongside stalling. These range from whole and fragmented pottery (i.e. a miniature vessel found in stratigraphic unit US 456F of structure F–see Fig 5A), and burnt bone fragments, to peculiar finds such as parts of woven basket, a weaving sword, a bone pin and loom weights [77] found in stratigraphic unit 619 of structure E (see Fig 5C–another bone pin was recovered in the same structure in a unit not analysed in this article). In the same unit (619—Fig 5C) of structure E two arrowheads made of bone were also found. One more bone arrowhead comes from structure F (stratigraphic unit 500—Fig 5B). Also the production of tar, well documented in structure F (stratigraphic unit 561—Fig 5A), points to a variety of domestic activities, given the vast range of uses of this substance (see above). The juxtaposition of *in situ* herbivore dung, domestic finds, and hearths allows us to conclude that structures E and F were not just byres or stables, but rather byre-houses. The possible goat/sheep fecal pellets documented in SMT 2a (Fig 7B) [69] suggests that ovicaprids were most likely the species that shared the domestic space with people. A preliminary assessment of the faunal assemblage (total NISP = 417) corroborates this hypothesis, as ovicaprids are the most represented species at Oppeano 4D (29% of the whole assemblage—Rosalind Gillis, pers. comm. 2021). This picture is in accordance with the subsistence economy of northern Italian Bronze Age communities. In Veneto, Trentino Alto Adige, and Emilia-Romagna regions faunal assemblages are in fact dominated by goat/sheep and cattle, mainly exploited for secondary products, meat, and–in the case of cattle–as draught animals [78–80].

The chronology of the Oppeano byre-houses (MBA 1, 1650–1550 cal a BCE) fits in the framework of the mobile herding strategy hypothesized for MBA communities of the Alpine area and Po plain [see 81–83]. The spread during the MBA of fortified hilltop sites (“castellieri”), interpreted as intermediate stations along the transhumance paths [84–86], also took place on the Lessini mountains, located just 50 km north of Oppeano [42, 86] (Fig 1). Systematic surveys along the main pathways connecting the Lessini high pastures to the Adige valley revealed numerous undated pastoral structures (i.e., sheepfolds, shelters, herdsman’s houses), and MBA bronze artifacts [87]. Carrer and Migliavacca [85] suggest that the Lessini plateaux, between 1,300 m and 1,800 m of altitude, were seasonally exploited in the MBA, probably by the communities in the plains south of Verona. This hypothesis is based on the similarity in material culture and on the geographical proximity between the two areas [88]. Even if a direct relationship between Bronze Age pastoralism and medieval or modern practices cannot be established with certainty [82], historical and ethnographic sources suggest movement of flocks between the Lessini mountains and the plain south of Verona since at least the Middle Ages [89]. With this evidence in mind, isotopic analyses on the M2 and M3 molars of ovicaprids and bovines, and pollen analysis on the dung, will be used to understand if the byre-houses of Oppeano were used only during certain periods of the year along transhumance paths connecting the Lessini mountains with the Adige floodplain in the MBA.

The use of wood ash and of combustion by-products on the living surfaces testified by SMT 1 and sub-types represents a floor maintenance practice that has been documented in other archaeological [34, 90–92] and ethnographic contexts in Europe [93]. The repetition of this practice multiple times in both structures suggests that the inhabitants consciously used ashes to keep the floor surface dry [93] or for hygienic purposes (i.e., killing parasites) [94]. The need to maintain the domestic space as “clean” and dry as possible appears coherent with byre-houses, since the close confinement of stock promotes high levels of parasite infestation [95]. The internal accretion sequences do not appear to have been cut or dug out to regain headroom as the floor level “grew” [96, 97]. The hearths kept being re-built on the same spot several times, floors kept growing, and new structures were rebuilt on top of former deposits. Micromorphology showed that all the sediments inside structures E and F accumulated in a protected (i.e., roofed) space, as no indicators of surface runoff or slacking were identified [65, 98, 99]. Moreover, the perfect preservation of finely laminated sediments indicates the absence of poaching in muddy conditions [64]. Laminations also survived thanks to the absence of bioturbation and to rapid burial. This is also suggested by the preservation of fragile components such as ash rhombs [62] and dung spherulites [100].

The production of wood tar resulted in a round patch of blackish material resembling the shape of the several hearths that were excavated at Oppeano. This simple arrangement is in line with recent experimental findings on tar production [101, 102], and was corroborated by organic petrology in SMT 3. Wood tar could be related to several activities carried out in the domestic space, some of which linked to animal husbandry. Wood tar is in fact primarily a form of glue [103, 104], but it can also protect vegetal remains from fungal attack [105], and treat a variety of human diseases thanks to its antiseptic properties [106, 107]. The zootechnical and ethnoarchaeological literature indicates that it played an important role in husbandry practices until recent times [108]. It was in fact used to treat also domestic animal diseases [106, 109–112] and to repel parasites [113, 114] and insects [115, 116].

**Fig 14:**
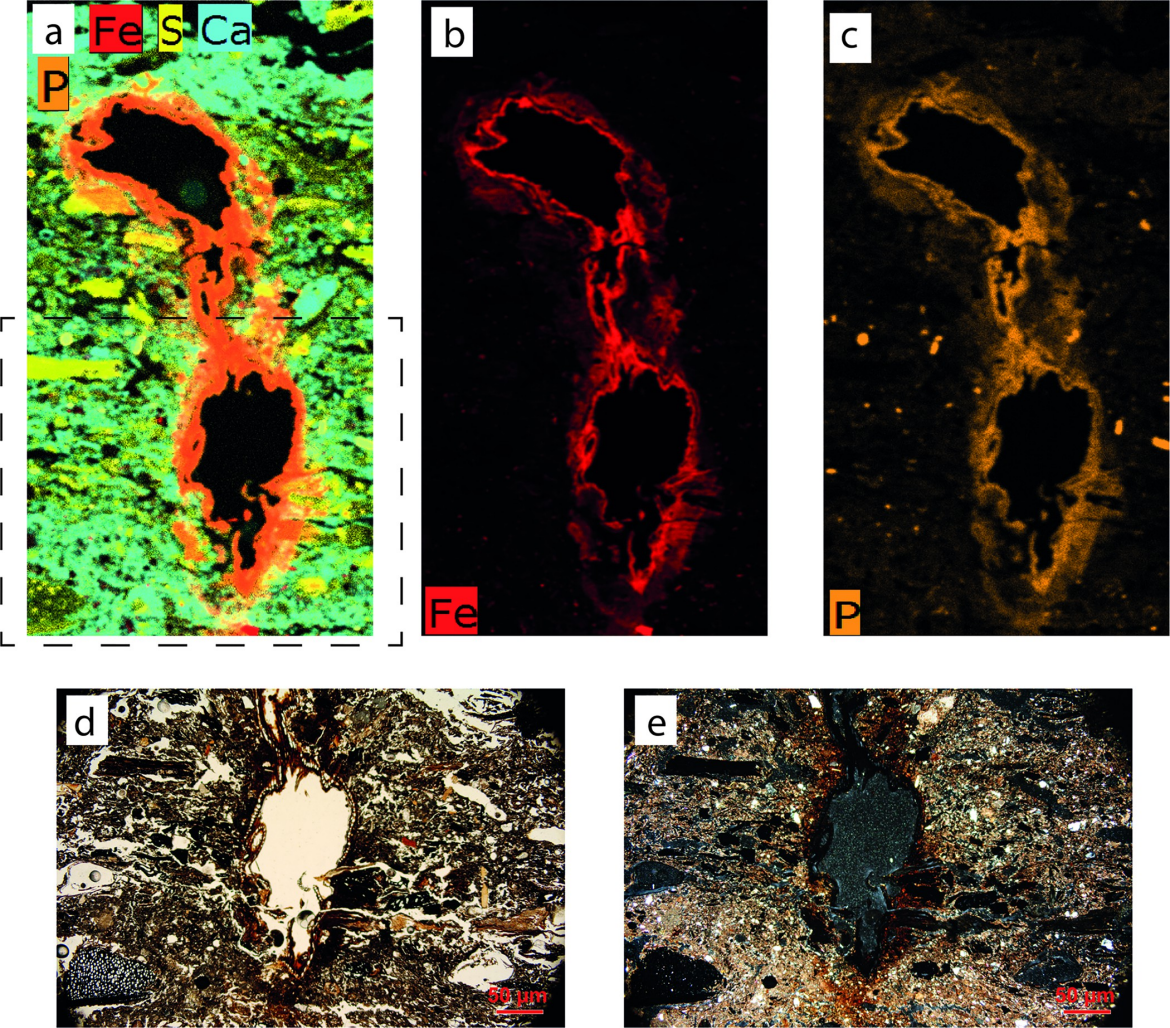
Fe- and P-rich hypocoating around a root channel. (a) multi-elemental μXRF map showing the abundance of Fe, S, Ca, and P. The dashed rectangle indicates the area shown in (d) and (e); (b, c) Same as (a), showing only the distribution of Fe and P respectively; (d) Microphotograph showing a detail of the root channel and of the hypocoating associated to it (PPL); (e): same as (d), XPL.

Micro-XRF on uncovered thin sections showed that sulphur is a key element to distinguish between ash and dung sub-units. Despite the role attributed in the literature to phosphorus in herbivore dung deposits [117], its signal in the SMT 2 sub-units varies from very low to nearly absent, while it is very abundant in association with calcium in all ashy sub-units [118] (Fig 8C and 8E). The low P content of herbivore dung is most likely related to the low protein intake with respect to carnivore-omnivores (similarly, for example, to the difference between fruit and insectivorous bat guano [119]). This difference and has also been determined analytically in comparative studies on present-day dung [120, 121]. Moreover, it is possible that P-bearing compounds migrated from the dung-rich sub-units, as suggested by the presence of phosphate nodules [122], of P-rich hypocoatings around rare roots channels (Fig 14), and by P absorption by charcoal fragments [123]. High S values, instead, together with silica from phytoliths (Si(OH)_4_; see [124]), characterized the chemical signature of herbivore dung in our deposits (Fig 8B and 8D). The strong S signal is related to the fresh (i.e., neither strongly decayed, nor burnt) nature of the organic matter in dung and to the activity of S-reducing microorganisms that act in wetlands [125]. The μXRF results show the potential of this technique in deciphering formation processes in wetland archaeological contexts. To the authors’ knowledge, no μXRF patterns are yet available from sites of this kind.

## Conclusion

Two structures exposed at the MBA 1 site of Oppeano 4D were interpreted as byre-houses thanks to the high-resolution study of their internal stratifications. As these structures were only partially intercepted by the excavation trench, we cannot establish if animals occupied also the unexcavated portions. However, the concept of “byre-house” (maison-etables or wohnstallhäuser respectively in French and German) applies to all living solutions involving humans and animals housed under the same roof [10]. This organization of the domestic space has been ethnographically documented in modern and contemporary societies. There are examples of people and animals living in the same room (i.e., Kabylie society; see [126]) or under one roof but in separate spaces (i.e., England, Germany, and Ireland; see respectively [11, 12, 127]). The term “byre-houses” therefore well applies to the MBA structures of Oppeano 4D. Under their roofed space, herbivore–probably ovicaprid–dung was trampled, and wood ash was regularly spread possibly for sanitary purposes. Hearth preparations were re-plastered several times on the same spot using local alluvial silty sands. Periodic reflooring was carried out with local geogenic materials as well. The exceptional preservation conditions of these byre-houses and of their internal stratifications derives from concomitant factors, such as: (a) The fast rate of accretion, which outpaced bioturbation; (b) The fact that the site was rapidly “engulfed” by peat and gyttja still within the MBA 1 time period; (c) The rise of the groundwater table in the MBA 1, and the post-abandonment deep burial of the site under Adige overbank sediments, both consequences of its geomorphological setting. These conditions allowed us to observe that the human activities within these byre-houses had resulted in the formation of finely stratified or even laminated sequences. This sedimentation style reflects a series of repetitive actions (see Leroi-Gourhan’s concept of *gesture* in [128] or of *habitus* in [129]). This cyclicity could never be identified in bioturbated and non-waterlogged contexts, where highly homogenized and complex “cultural layers”, “anthropic horizons”, or “dwelling soils” tend to form (translation of the French *sols d’occupation* and of the Italian *suoli d’abitato–*see [130–133]) This type of deposit is encountered in northern Italian Bronze Age sites (and in several others) that did not experience the peculiar taphonomic conditions of Oppeano 4D [134].

It remains to be fully understood what the role of the Oppeano 4D byre-houses was in the MBA 1 economy of this area. The reasons behind byre-houses are a debated issue in European archaeology [135]. Zimmermann [136] denies that housing animals warms up the domestic space, this practice more frequently resulting in unhealthy and humid conditions. Nisly [11] states that stalled animals prefer lower temperatures than humans, and that in winter shelter from wind and dry bedding are the only requirements. Social or ideological aspects behind the choice of the byre-house model cannot be ruled out [11, 137, 138]. In the “pastoral ideology” that characterized northern Europe in the Bronze Age [139], for example, livestock represented a status symbol and a precious asset [140–142]. Isotopic and pollen analyses will hopefully clarify if these byre-houses really represented a temporary station along the herding paths that connected the Adige plain with the Lessini mountains. If this was not the case, flocks probably exploited year-round the pastures in the immediate vicinity of the site, on the well-drained rubified Luvisols/Alfisols of the Adige alluvial fan [143, 144].

High-resolution sediment analysis is currently being used to investigate if the remaining seven structures unearthed at Oppeano were also byre-houses or had other functions. Identifying different day-to-day domestic activities ([145]; see also [146]) performed in different dwelling units (*sensu* [147]), would suggest a certain degree of social differentiation and complexity. The geoarchaeological study of domestic deposits can therefore complement the study of the size and shape of houses, their variability, and their distribution within a settlement. It would greatly complement those aspects that until now have been employed to infer the social organization and demography of Bronze Age communities [148, 149] and their position with respect to landscape units [150].

## Supporting information

S1 Table Archaeological structure interpreted as byre-houses in Europe (Neolithic-Middle Ages). Archaeological sites where Neolithic to Middle Age byre-houses were identified (the relative chronology of each site refers to the one published in “References”). “Key features” indicates the proxies employed to identify the structures as byre-houses. Proxies are numbered as follows: 1) Architectural elements (see below); 2) Animal dung; 3) Animal bones; 4) Finds; 5) High P concentration; 6) Staining; 7) Cattle hoof prints; 8) Presence of a sunken floor area with organic infill; 9) Coexistence of hearth and macroscopically observed dung layers; 10) Micromorphology; 11) Macrofossils (i.e., plant remains, seeds, insects); 12) Loss-on-ignition. Following Waterbolk [151], the label “architectural elements” (number 1 in the column “Key Features”) synthetizes several features that are: transversal or longitudinal partitions, separating single or double “boxes” for the animals; structural elements typically observed in byres (i.e., thinner and shallower extra posts in the line of the roof-bearing uprights; regular dense spacing of upright pairs); ditch in the longitudinal axis of the structure to collect manure; stone floors or evidence for matting; extra posts placed at regular distances near the side wall, possibly serving for fixing the heads of the animals with a rope; door at one of the short sides of the structure.
(DOCX)

S2 Table Radiocarbon dates related to the phase 2 of the settlement Oppeano 4D. Asterisks mark the dates coming from structures E and F, studied here (see Figs 2 and 5).
(DOCX)

S1 Appendix PPL and XPL scans and interpreted version (SMT) of the studied thin sections. For each thin section, the different scans (PPL and XPL), the subdivision in sub-units, and the interpreted version are available as overlapping layers that can be turned on and off using a PDF file reader. For a correct visualization, Adobe Acrobat is recommended. If one prefers a browser visualization, the plug-in Adobe PDF reader (free download) offers the same a correct visualization. Information on how to install and use the plug-in Adobe PDF reader can be find at this link: https://helpx.adobe.com/acrobat/using/display-pdf-in-browser.html (updated on May 4^th^, 2022). In Adobe PDF software and plug-in, the “layers” panel can be found and works as it follows:
Choose View > Show/Hide > Navigation Panes > Layers.To hide a layer, click the eye icon. To show a hidden layer, click the empty box (a layer is visible when the eye icon is present, and hidden when the eye icon is absent).From the options menu, choose one of the following: List Layers For All Pages. Additional information can be found at this link: https://helpx.adobe.com/acrobat/using/pdf-layers.html (updated on May 4^th^, 2022).
(PDF)

S1 Dataset (XLSX)

S1 File (R)
