# Supplementary material for: High-resolution sediment analysis reveals Middle Bronze Age byre-houses at the site of Oppeano (Verona province, NE Italy)
Source: PLoS One. 2022 Aug 31;17(8):e0272561. doi: 10.1371/journal.pone.0272561 (PMC9432763; doi:10.1371/journal.pone.0272561)
Supplement: S1 Table — Archaeological sites where Neolithic to Middle Age byre-houses were identified (the relative chronology of each site refers to the one published in “References”). “Key features” indicates the proxies employed to identify the structures as byre-houses. Proxies are numbered as follows: 1) Architectural elements (see below); 2) Animal dung; 3) Animal bones; 4) Finds; 5) High P concentration; 6) Staining; 7) Cattle hoof prints; 8) Presence of a sunken floor area with organic infill; 9) Coexistence of hearth and macroscopically observed dung layers; 10) Micromorphology; 11) Macrofossils (i.e., plant remains, seeds, insects); 12) Loss-on-ignition. Following Waterbolk [151], the label “architectural elements” (number 1 in the column “Key Features”) synthetizes several features that are: transversal or longitudinal partitions, separating single or double “boxes” for the animals; structural elements typically observed in byres (i.e., thinner and shallower extra posts in the line of the roof-bearing uprights; regular dense spacing of upright pairs); ditch in the longitudinal axis of the structure to collect manure; stone floors or evidence for matting; extra posts placed at regular distances near the side wall, possibly serving for fixing the heads of the animals with a rope; door at one of the short sides of the structure. (DOCX) [file pone.0272561.s001.docx]

**S1 Table**

| **Site name** | **Country** | **House type** | **Key features** | **Relative chronology** | **References** |
| --- | --- | --- | --- | --- | --- |
| Altwies | Luxemburg | Three-aisled long house | 1, 5 | Neolithic | [1] |
| Pestenacker | Germany | ? | 1, 2 | Neolithic | [2] |
| Straubing-Öberau | Germany | Two-aisled long house | 1 | Neolithic | [3,4] |
| Flögeln-Eekhöltjen | Germany | Two-aisled long house | 1, 5 | Late Neolithic | [5] |
| Vinge | Germany | Two-aisled long house | 6 | Late Neolithic | [6] |
| Kongehøj | Denmark | Three-aisled long house | 1 | Early Bronze Age | [7–9] |
| Landlyst | Denmark | Three-aisled long house | 1 | Early Bronze Age | [8] |
| Legård (site n. 110112-279) | Denmark | Three-aisled long house | 1 | Early Bronze Age | [8,10] |
| Spjald (site n. 180401-204) | Denmark | Three-aisled long house | 1 | Early Bronze Age | [8,11,12] |
| Mannehøjgård | Denmark | Three-aisled long house | 1 | Early Bronze Age | [8,9,13] |
| Dalsgård (site n. 170805-307) | Denmark | Three-aisled long house | 1 | Early Bronze Age | [9,14] |
| Enderupskov | Denmark | Three-aisled long house | 1 | Early Bronze Age | [8,9,15] |
| Gilmosevej (site n. 180318-57) | Denmark | Three-aisled long house | 1 | Early Bronze Age | [8,9,16] |
| Hesel | Germany | Two-aisled long house | 1 | Early Bronze Age | [4,17] |
| Noordwijk-Bronsgeest | Netherlands | Three-aisled long house | 3, 4 | Early Bronze Age | [18] |
| Limensgård | Denmark | Two-aisled long house | 1 | Early Bronze Age | [3,4] |
| Elp | Netherlands | Three-aisled long house | 1 | Middle Bronze Age | [19,20] |
| Enspijk | Netherlands | Three-aisled long house | 1 | Middle Bronze Age | [4,21] |
| Angelslo-Emmerhout | Netherlands | Three-aisled long house | 1, 6 | Middle Bronze Age | [20,22,23] |
| Loon op Zand | Netherlands | Three-aisled long house | 1 | Middle Bronze Age | [12,24,25] |
| Gram | Denmark | Three-aisled long house | 1 | Middle Bronze Age | [4,26] |
| Nybro | Sweden | Two-aisled long house | 1 | Late Bronze Age | [8,27,28] |
| Snebæk (site n. 130107-292) | Denmark | Three-aisled long house | 1 | Late Bronze Age | [8,9,29] |
| Regteren | Netherlands | Three-aisled long house | 1 | Bronze Age | [4,30] |
| Bjerre 2, 4 | Denmark | Three-aisled longhouse | 1 | Bronze Age | [8,9,31,32] |
| Dalen | Netherlands | Three-aisled long house | 1 | Bronze Age | [23] |
| Nijnsel | Netherlands | Four-aisled long house | 1 | Bronze Age | [33] |
| Ringkøbing | Denmark | Three-aisled long house | 1 | Bronze Age | [34] |
| Telgte | Germany | Four-aisled long house | 1 | Bronze Age | [35] |
| Zijderveld | Netherlands | Three-aisled long house | 1 | Bronze Age | [4,36] |
| Nygårdstoft | Denmark | Three-aisled long house | 1 | Bronze Age | [8,37] |
| Højgård | Denmark | Three-aisled long house | 5 | Bronze Age | [8,15] |
| Jemgumkloster | Germany | Three-aisled long house | 1 | Iron Age | [38] |
| Oldendorp | Germany | Three-aisled long house | 1 | Iron Age | [38] |
| Jemgum | Germany | Three-aisled long house | 1 | Iron Age | [38] |
| Boomborg-Hatzum | Germany | Three-aisled long house | 1 | Iron Age | [38,39] |
| Aleburg - Befort | Luxemburg | Three-aisled long house | 1 | Iron Age | [40] |
| Assendelver Polder - Site Q | Netherlands | Three-aisled longhouse | 1, 2 | Iron Age | [41] |
| Zijderveld | Netherlands | Two-aisled long house | 1 | Iron Age | [36] |
| Ezinge | Netherlands | Three-aisled long house | 1 | Iron Age | [20,42] |
| Hijken | Netherlands | Three-aisled long house | 1 | Iron Age | [20] |
| Vlaardingen-site 37-oosT-22 | Netherlands | Two/three-aisled long house | 9 | Iron Age | [43] |
| Vlaardingen – De Vergulde Hand West | Netherlands | Two/three-aisled long house | 1, 2, 11 | Iron Age | [44,45] |
| Vlaardingen-Holierhoeksepolder | Netherlands | Three-aisled long house | 1, 2 | Iron Age | [43,46] |
| Haps | Netherlands | Two-aisled long house | 1 | Iron Age | [46,47] |
| Köpinge 21:2 | Sweden | Three-aisled long house | 1 | Iron Age | [48] |
| Sønderris | Sweden | Three-aisled long house | 1, 12 | Iron Age | [49] |
| Myrthue | Denmark | Long house | 1 | Iron Age | [50] |
| Nørre Snede | Denmark | Long house | 5 | Iron Age | [51] |
| Grøntoft | Denmark | Three-aisled long house | 1 | Iron Age | [20] |
| Østerbølle | Denmark | Long house | 1 | Iron Age | [52] |
| Gronheden | Denmark | Long house | 1 | Iron Age | [52] |
| Heltborg | Denmark | Three-aisled long house | 1 | Iron Age | [52,53] |
| Blidstrup Mark | Denmark | Long house | 1 | Iron Age | [52] |
| Kildevej | Denmark | Long house | 1 | Iron Age | [52] |
| Lynggård | Denmark | Long house | 1 | Iron Age | [52] |
| Pigens Vadested | Denmark | Two-aisled long house | 1 | Iron Age | [52] |
| Siggård | Denmark | Long house | 8 | Iron Age | [52] |
| Hesselagergård | Denmark | Long house | 8 | Iron Age | [52] |
| Højgård | Denmark | Long house | 1 | Iron Age | [52] |
| Flejsborg | Denmark | Long house | 1, 3 | Iron Age | [54,55] |
| Skørbaek | Denmark | Long house | 1 | Iron Age | [52] |
| Års Mark | Denmark | Long house | 1 | Iron Age | [52] |
| Solbjerg | Denmark | Long house | 1, 3, 8 | Iron Age | [52,55] |
| Ginderup | Denmark | Long house | 1, 3, 8 | Iron Age | [55–57] |
| Siggård | Denmark | Long house | 1, 2, 3 | Iron Age | [55,58] |
| Engeistrup | Denmark | Long house | 1, 5 | Iron Age | [52] |
| Klegod | Denmark | Long house | 1 | Iron Age | [52] |
| Lonne Hede | Denmark | Three-aisled long house | 1, 5 | Iron Age | [52,59] |
| Vildbjerg | Denmark | Long house | 1 | Iron Age | [52] |
| Nørre Tranders | Denmark | Three-aisled long house | 1, 3, 8 | Iron Age | [55,60] |
| Hol østre | Norway | Three-aisled long house | 1 | Iron Age | [61] |
| Feddersen Wierde | Germany | Three-aisled long house | 1 | Roman Age | [38,62] |
| Flögeln-Eekhöltjen | Germany | Three-aisled long house | 1, 5 | Roman Age | [5,38] |
| Vorbasse | Denmark | Three-aisled long house | 1 | Roman Age | [38] |
| Sjaelborg | Denmark | Three-aisled long house | 1 | Roman Age | [20,22] |
| Alphen-Ekeren | Belgium | Two-aisled long house | 7, 8 | Roman Age | [63] |
| Brecht-Zoegweg | Belgium | Two-aisled long house | 8, 10 | Roman Age | [64,65] |
| Lieshout-Beekseweg West | Netherlands | Two-aisled long house | 8 | Roman Age | [63] |
| Mierlo-Hout-Brandevoort | Netherlands | Two-aisled long house | 8 | Roman Age | [63] |
| Hoogeloon | Netherlands | Two-aisled long house | 8 | Roman Age | [66–68] |
| Weert-Kampershoek Noord | Netherlands | Two-aisled long house | 8 | Roman Age | [68] |
| Weert-Molenakker | Netherlands | Two-aisled long house | 8 | Roman Age | [63] |
| Nederweert-Rosveld | Netherlands | Two-aisled long house | 8 | Roman Age | [63] |
| Tongres - Kielenstraat | Belgium | Two-aisled long house | 1, 2, 8 | Roman Age | [69] |
| Oosterhout | Netherlands | Two/three-aisled long house | 1 5, 7, 8, 11 | Roman Age | [70,71] |
| Riethoven-Heesmortel | Netherlands | Two-aisled long house | 8 | Roman Age | [63] |
| Deurne-Groot Bottelsche Akkers | Netherlands | Two-aisled long house | 8 | Roman Age | [63] |
| Fochteloo | Netherlands | Three-aisled long house | 1 | Roman Age | [20] |
| Wijster | Netherlands | Three-aisled long house | 1 | Roman Age | [20,38] |
| Ghent-Kluizendok | Belgium | Two-aisled long house | 1, 8 | Roman Age | [72,73] |
| Aalter-Langevoorde | Belgium | ? | 8 | Roman Age | [73,74] |
| Rogaland | Norway | Three-aisled long house | 1 | Late Iron Age | [75] |
| Røyksund | Norway | Three-aisled long house | 1 | Viking Age | [76] |
| Moi I/V | Norway | Three-aisled long house | 1 | Viking Age | [76] |
| Borg | Norway | Three-aisled long house | 1, 5, 11 | Viking Age | [77,78] |
| Helganeset | Norway | Long house | 1 | Viking Age | [76] |
| Aðalstræti 16 | Iceland | Three-aisled long house | 10 | Viking Age | [79] |
| Gerlingen | Germany | Pit house | 1, 8 | Early Medieval | [80,81] |
| Leens | Netherlands | Three-aisled long house | 1 | Early Medieval | [20] |
| Odoorn | Netherlands | Three-aisled long house | 1 | Early Medieval | [20,38] |
| Pitcarmick | Scotland | Three-aisled long house | 1 | Early Medieval | [82] |
| Elisenhof | Germany | Three-aisled long house | 1, 2 | Medieval | [38] |
| Wurt Niens - Nordenham | Germany | Three-aisled long house | 1 | Medieval | [38] |

**References in S1 table**

1. Fechner K, De Lil A, Clavel V, Hus J, Teheux E, Broes F, et al. Cartographie du phosphore dans des bâtiments allongés du Néolithique dans le Nord de la France, en Belgique et au Luxembourg. Rev archéologique Picardie Numéro spécial. 2011;28: 275–298. doi:10.3406/pica.2011.3334

2. Schönfeld G. Ein Wohnstallhaus aus der jungneolithischen Talbodensiedlung von Pestenacker. Das archäologish Jahr Bayern 1991. 1991; 44–50.

3. Nielsen PO. Limensgård and Grødbygård Settlements with house remains from the Early, Middle and Late Neolithic on Bornholm. In: Fabech C, Ringtved J, editors. Settlement and Landscape: Proceedings of a Conference in Aarhus, Denmark, May 4-7 ·1998. Højbjerg: Jutland Archaeological Society; 1999. pp. 149–165.

4. Arnoldussen S. A living landscape. Bronze Age settlement sites in the Dutch river area (c. 2000-800 BC). Leiden: Sidestone Press; 2008.

5. Zimmermann WH. Phosphate mapping of a Funnel Beaker Culture house from Flögeln-Eekhöltjen, district of Cuxhaven, Lower Saxony. In: Fokkens H, Coles BJ, Van Gijn AL, Kleijne JP, Ponjee HH, Slappendel CG, editors. Between foraging and farming An extended broad spectrum of papers presented to Leendert Louwe Kooijmans. Leiden: Leiden University Press; 2008. pp. 123–129.

6. Johannsen JW. Mansion on the hill – A monumental Late Neolithic house at Vinge, Zealand, Denmark. J Neolit Archaeol. 2017;19: 1–28. doi:10.12766/jna.2017.1

7. Poulsen ME. Bygherrerapport for HBV j. nr. 1275. Kongehøj Etape II. Arkæologiske undersøgelser af bebyggelse fra bondestenalder og bronzealder. Brørup; 2008.

8. Bech J-H, Olsen A-LH. Early Bronze Age houses from Thy, Northwest Denmark. In: Willroth K-H, editor. Siedlungen der älteren Bronzezeit Beiträge zur Siedlungsarchäologie und Paläoökologie des II.vorchristichen Jahrtausends in Südskandinavien, Norddeutschland und den Niederlanden. Neumünster: Wachholtz Verlag; 2013. pp. 9–32.

9. Bech J-H, Rasmussen M. Thy and the outside world in the Bronze Age. Regional variations in a North Sea perspective. In: Bech J-H, Eriksen BV, Kristiansen K, editors. Bronze Age Settlement and Land-Use in Thy, Northwest Denmark. Højbjerg: Jutland Archaeological Society; 2018. pp. 25–106.

10. Mikkelsen M, Kristiansen K. Legaard. In: Bech J-H, Eriksen BV, Kristiansen K, editors. Bronze Age Settlement and Land-Use in Thy, Northwest Denmark. Højbjerg: Jutland Archaeological Society; 2018. pp. 505–538.

11. Rasmussen M, Adamsen C. Settlement. In: Hvass S, Storgaard B, editors. Digging into the past: 25 years of archaeology in Denmark. Højbjerg, København: Aarhus University Press; 1993. pp. 136–141.

12. Fokkens H. Cattle and martiality. Changing relations between man and landscape in the Late Neolithic and the Bronze Age. In: Fabech C, Ringtved J, editors. Settlement and Landscape: Proceedings of a Conference in Aarhus, Denmark, May 4-7 ·1998. Højbjerg: Jutland Archaeological Society; 1999. pp. 35–43.

13. Laursen ST. HBV 1212 Mannehøjgård. Bygherrerapport for den arkæologiske undersøgelse HBV 1212 Mannehøjgård. Brørup; 2005.

14. Jeppesen J. En bebyggelse fra ældre bronzealder ved Dalsgaard. Ca. 1750–1000 f. Kr. In: Gyldion A, Jeppesen J, Lindbloom C, editors. Oldtiden på vej mellem Riis og Ølholm. Vejle: Vejle Museum; 2004. pp. 43–46.

15. Ethelberg P. Bronzealderen. In: Ethelberg P, Jørgensen E, Meier D, Robinson D, Hardt N, editors. Det sønderjyske landbrugs historie Sten- og Bronzealder. Haderslev: Haderslev Museum; 2000. pp. 135–280.

16. Pedersen VJ. Gilmosevej. Foreløbig analyse af bopladsspor fra yngre stenalder og bronzealder. Herning Museum Midtjyske Fortællinger. 2006; 27–34.

17. Schwarz W. Bronzezeitliche Hausgrundrisse von Hesel im Landkreis Leer. Die Kd N F. 1996;21: 21–50.

18. Van der Velde HM. The Early Bronze Age farmstead of Noordwijk. In: Arnoldussen S, Fokkens H, editors. Bronze Age settlement sites in the Low Countries. Oxford: Oxbow Books; 2008. pp. 163–170.

19. Waterbolk HT. The Bronze Age settlement of Elp. Helinium. 1964;4: 97–131.

20. Waterbolk HT. Evidence of cattle stalling in excavated pre- and protohistoric houses. In: Clason AT, editor. Archaeozoological Studies Papers of the Archaeozoological Conference 1974, held at the Biologisch-Archaeologisch Instituut of the State University of Groningen. Amsterdam: North-Holland Publishing Company; 1975. pp. 383–394.

21. Ter Wal A. Verbreding A2 Everdingen - Deil Vindplaats 2. Deventer: Bouwhistorie, Archeologie, Architectuur- en Cultuurhistorie; 2005. doi:10.17026/dans-235-54a6

22. Thomsen N. Hus og kælder i romersk jernalder. KUML. 1959; 13–27.

23. Kooi P. Bronze Age settlements in Drenthe. In: Arnoldussen S, Fokkens H, editors. Bronze Age Settlements in the Low Countries. Oxford: Oxbow Books; 2008. pp. 59–68.

24. Roymans N, Hiddink H. Nederzettingssporen uit de bronstijd en de vroege ijzertijd op de Kraanvensche Heide te Loon op Zand. In: Fokkens H, Roymans N, editors. Nederzettingen uit de bronstijd en de vroege ijzertijd in de Lage Landen. Amersfoort: Rijksdienst voor het Oudheidkundig Bodemonderzoek; 1991. pp. 111–127.

25. Fokkens H. The periodisation of the Dutch Bronze age: A critical review. In: Butler JJ, Metz WH, Beek BL van, Steegstra H, editors. Patina Essays presented to Jay Jordan Butler on the occasion of his 80th birthday, Groningen/Amsterdam. Groningen, Amsterdam: Metz, Van Beek & Steegstra; 2001. pp. 241–262.

26. Ethelberg P. Brdr. Gram. Arkæologiske udgravninger i Danmark. 1995;1994: 213.

27. Nielsen J, Mikkelsen M. Nybro: en grav fra yngre stenalder og en boplads fra yngre bronzealder. Mark og montre. 1985;21: 55–62.

28. Jensen J. Bronze Age Research in Denmark 1970–1985. J Danish Archaeol. 1987;6: 155–174. doi:10.1080/0108464x.1987.10589984

29. Bertelsen JB, Christensen M, Mikkelsen M, Mikkelsen P, Nielsen J, Simonsen J. Bronzealderens bopladser i Midt- og Nordvestjylland. Skive: Skive Museum; 1996.

30. Verlinde AD. Rechteren, Gem. Dalfsen. In: Verlinde AD, editor. Archeologische Kroniek van Overijssel over 1980/1981. Zwolle: Waanders Uitgeverij Waanders; 1982. pp. 182–185.

31. Bech J-H. Bronze Age settlements on raised sea-beds at Bjerre, Thy, NW-Jutland. In: Anendorp JJ, editor. Forschungen zur bronzezeitlichen Besiedlung in Nord- und Mitteleuropa: Internationales Symposium vom 9-11 Mai 1996 in Hitzacker. Espelkamp: Verlag Marie Leidorf GmbH; 1997. pp. 3–15. Available: http://scholar.google.com/scholar?hl=en&btnG=Search&q=intitle:Bronze+Age+settlements+on+raised+sea-beds+at+Bjerre,+Thy,+NW-Jutland#0

32. Mikkelsen DK, Bech J-H. Bjerre 4 - settlement, cemetery and field system. In: Bech J-H, Eriksen BV, Kristiansen K, editors. Bronze Age Settlement and Land-Use in Thy, Northwest Denmark. Højbjerg: Jutland Archaeological Society; 2018. pp. 133–149.

33. Beex G, Hulst RS. A Hilversum-culture settlement near Nijnsel, municipality of St. Oedenrode, North-Brabant. Ber van Rijksd voor het Oudheidkd Bodemonderz. 1968;18: 117–129.

34. Becker CJ. Siedlungen der Bronzezeit und der vorrömischen Eisenzeit in Dänemark. Offa Berichte und Mitteilungen zur Urgeschichte, Frühgeschichte und Mittelalterarchäologie. 1982;39: 53–71.

35. Reichman C. Ein Bronzezeitliches Gehòft bei Telgte, Kr. Wahrendorf. Archäologisches Korrespondenzblatt. 1982;12: 437–449.

36. Hulst RS. A contribution to the study of Bronze Age and Iron Age house-plans: Zijderveld. Ber van Rijksd voor het Oudheidkd Bodemonderz. 1975;23 (1973): 103–107.

37. Feveile C, Lauridsen J. ASR 1820 Nygårdstoft – bebyggelse fra bronzealderen og fra nyere tid. Ribe; 2003.

38. Haarnagel W. Hausbau. In: Kossack G, Behre K, Schmid P, editors. Archäologische und naturwissenschaftliche Untersuchungen an ländlichen und frühstädtischen Siedlungen im deutschen Küstengebiet vom 5 Jahrhundert v Chr bis zum 11 Jahrhundert n Chr. Weinheim: Acta Humaniora; 1984. pp. 167–193.

39. Behre K-E. Landwirtschaftliche Entwicklungslinien und die Veränderung der Kulturlandschaft in der Bronzezeit Europas. In: Hänsel B, editor. Mensch und Umwelt in der Bronzezeit Europas = Man and Environment in European Bronze Age. Kiel: Oetker-Voges Verlag; 1998. pp. 91–109.

40. Schindler R. Die Aleburg von Befort in Luxemburg. Hemecht. 1969;1: 37–50.

41. Therkorn LL, Brandt RW, Pals P, Taylor M. An Early Iron Age Farmstead: Site Q of the Assendelver Polders Project. Proc Prehist Soc. 1984;50: 351–373. doi:10.1017/S0079497X0000757X

42. Van Griffen AE. Der Warf in Ezinge, Provinz Groningen, Holland, und seine westgermanischen Häuser. Ger Anzeiger der Römisch-Germanischen Kommission des Dtsch Archäologischen Instituts. 1936;20: 40–47.

43. Van Heeringen RM. The Iron Age in the Western Netherlands III: Site catalogue and pottery description, map sheet I. Ber van Rijksd voor het Oudheidkd Bodemonderz. 1987;37: 39–121.

44. Eijskoot Y, Brinkkemper O, De Ridder T. Vlaardingen- De Vergulde Hand-West. Onderzoek van archaeologische resten van de middenbronstijd tot en met de late middeleeuwen. Amersfoort: Rijksdienst voor het Cultureel Erfgoed; 2011.

45. Braadbaart F, van Brussel T, van Os B, Eijskoot Y. Fuel remains in archaeological contexts: Experimental and archaeological evidence for recognizing remains in hearths used by Iron Age farmers who lived in peatlands. Holocene. 2017;27: 1682–1693. doi:10.1177/0959683617702231

46. Mathiot D. Les habitats ruraux des cinq derniers siècles avant notre ère entre le cours de la Somme et le delta Meuse-Rhin: Formes et organisations. Rev Nord. 2012;398: 7–26. doi:10.3917/rdn.398.0007

47. Verwers GJ. Das Kamps Veld in Haps Neolithicum, Bronzezeit und Eisenzeit. Analecta Praehist Leiden. 1972;5: 143–148.

48. Tesch S. Houses, farmsteads, and long-term change. A regional study of prehistoric settlements in the Köpinge area, in Scania, Southern Sweden. Uppsala University. 1993.

49. Grabowski R, Linderholm J. The use of space on two Early Iron Age house sites in South-West Jutland, South Scandinavia: A geoarchaeological multiproxy approach. J Archaeol Sci Reports. 2022;42: 103405. doi:10.1016/j.jasrep.2022.103405

50. Thomsen N. Myrthue, et gårdsanlæg fra jernalder. KUML. 1964;14: 15–30.

51. Zölitz R. Phosphatuntersuchungen zur funktionalen Differenzierung in einem všlkerwanderungszeitlichen Langhaus von Norre Snede, DŠnemark. Archäologisches Korrespondenzblatt. 1980;10: 273–280.

52. Webley L. Iron Age Households: Structure and Practice in Western Denmark, 500 BC—AD 200. Højbjerg: Jutland Archaeological Society; 2008.

53. Bech J-H. The Iron Age village mound at Heltborg, Thy. J Danish Archaeol. 1985;4: 129–146. doi:10.1080/0108464x.1985.10589942

54. Klingenberg S, Nielsen S. Amfora. Skalk. 1994;4: 3–8.

55. Nielsen JN. The burnt remains of a house from the Pre-Roman Iron Age at Nørre Tranders, Aalborg. In: Rasmussen M, editor. Iron Age houses in flames Testing house reconstructions at Lejre. Lejre: Historical-Archaeological Experimental Centre; 2007. pp. 16–31.

56. Kjær H. Enny Hustomt paa Oldtidsbopladsen ved Ginderup. Fra Natl Arb. 1930; 19–30.

57. Hatt G. Jernalderbopladsen ved Ginderup i Thy. Fra Natl Arb. 1935; 39–51.

58. Aabo Jørgensen C. Siggård. Arkæologiske udgravninger i Danmark Katalog 1994. 1995; 89.

59. Petersen L, Jansen CK. Fosfatundersøgelser på Kulturhistorisk Museum: spottest af ældre jernalders gårdsanlæg. Kult Museum Randers Årb. 1995; 90–112.

60. Nielsen JN. Flammernes bytte. Skalk. 2002;6: 5–10.

61. Helliksen W. Farms in transition: A study of settlement patterns in Eastern Norway, 300 B.C. to A.D. 1200. In: Rasmus Brandt J, Karlsson L, editors. From huts to houses Transformations of ancient societies Proceedings of an International Seminar organized by the Norwegian and Swedish Institutes in Rome, 21-24 September 1997. Stockholm: Paul Åströms Förlag; 2001. pp. 89–93.

62. Haarnagel W. Die Grabung Feddersen Wierde: Methode, Hausbau, Siedlungs- und Wirtschaftsformen sowie Sozialstruktur. Wiesbaden: Franz Steiner; 1979.

63. Hiddink H, Roymans N. Exploring the rural landscape of a peripheral region. In: Roymans N, Derks T, Hiddink H, editors. The Roman villa of Hoogeloon and the archaeology of the periphery. Amsterdam: Amsterdam University Press; 2015. pp. 45–86.

64. Delaruelle S, Verbeek C, De Clercq W. Wonen en leven op het HSL-traject in de Romeinse tijd (ca. 50 v.C.-476 n.C). In: Verbeek C, Delaruelle S, Bungeneers J, editors. Verloren voorwerpen Archeologisch onderzoek op het HSL-traject in de provincie Antwerpen. Antwerp: Provincieraad van Antwerpen; 2004. pp. 189–258.

65. Hinsch Mikkelsen J, Langhor R, Vanwesenbeeck V, Bourgeois I, De Clercq W. The byre’s tale. Farming nutrient-poor cover sands at the edge of the Roman Empire (NW-Belgium). In: Deák J, Ampe C, Hinsch Mikkelsen J, editors. Soils as records of past and Present From soil surveys to archaeological sites: research strategies for interpreting soil characteristics. Bruges: Raakvlak Archaeology, Monuments and Landscapes of Bruges and Hinterland; 2019. pp. 65–84. doi:10.5281/zenodo.3421029

66. Roymans N. The sword or the plough. Regional dynamics in the romanisation of Belgic Gaul and the Rhineland area. In: Roymans N, editor. From the sword to the plough: Three studies on the earliest romanisation of northern Gaul. Amsterdam: Amsterdam University Press; 1996. pp. 9–126.

67. Hiddink H. The villa settlement of Hoogeloon-Kerkakkers. In: Roymans N, Derks T, Hiddink H, editors. The Roman villa of Hoogeloon and the archaeology of the periphery. Amsterdam: Amsterdam University Press; 2015. pp. 87–124.

68. Roymans N, Derks T. Rural habitation in the area of the Texuandri (Southern Netherlands/Northern Belgium). In: Reddé M, editor. Gallia Rustica 1: “Les campagnes du nord-est de la Gaule, de la fin de l’âge du Fer à l’Antiquité tardive.” Bordeaux: Ausonius éditions; 2017. pp. 97–123. Available: https://hal.archives-ouvertes.fr/hal-03029626

69. Vanderhoeven A. The earliest urbanisation in Northern Gaul: some implications of recent research in Trongres. In: Roymans N, editor. From the sword to the plough: Three studies on the earliest romanisation of northern Gaul. Amsterdam: Amsterdam University Press; 1996. pp. 189–260.

70. Buurman J. Carbonised plant remains and phosphate analysis of two Roman period house plans with sunken byres at Oosterhout. Ber van Rijksd voor het Oudheidkd Bodermonderzoek. 1990;40: 285–296.

71. Van der Sanden W. The Ussen project: large-scale settlement archaeology of the period 700 BC-AD 250, a preliminary report. Analecta Praehist Leiden. 1987;20: 95–123.

72. De Clercq W, Laloo P, Perdaen Y, Crombé P. Grootschalig nederzettingsonderzoek in een inheems-Romeins landschap: het preventief archeologisch onderzoek “Kluizendokproject” in de Gentse haven (fase 2005-2006). Journée D’Archéologie Romaine –Romeinendag. 2007; 59–66.

73. De Clercq W. Roman rural settlements in Flanders. Perspectives on a ‘non-villa’ landscape in extrema Galliarum. In: Roymans N, Derks T, editors. Villa Landscapes in the Roman North. Amsterdam: Amsterdam University Press; 2011. pp. 235–258. doi:10.1515/9789048514830-012

74. De Clercq W, Mortier S. Aalter-Langevoorde. In: Bourgeois J, Bourgeois I, Cherretté B, editors. Bronze Age and Iron Age communities in North-Western Europe. Brussels: Vlaams Kennis- en Cultuurforum; 2003. pp. 193–195.

75. Bjørdal E. Late Iron Age settlement evidence from Rogaland. In: Iversen F, Petersson H, editors. The agrarian life of the North 2000 BC–AD 1000: Studies in rural settlement and farming in Norway. Cappelen Damm Akademisk; 2017. pp. 241–274.

76. Hem Eriksen M. Architecture, society, and ritual in viking age scandinavia: Doors, dwellings, and domestic space. Cambridge: Cambridge University Press; 2019. doi:10.1017/9781108667043

77. Arrhenius B, Freij H. Physical survey and soil analysis. In: Munch GS, Johansen OS, Roesdahl E, editors. Borg in Lofoten A chieftain’s farm in north Norway. Trondheim: Tapir Academic Press; 2003. pp. 77–85.

78. Herschend F, Mikkelsen DK. The main building at Borg (I:1). In: Munch GS, Johansen OS, Roesdahl E, editors. Borg in Lofoten A chieftain’s farm in north Norway. Trondheim: Tapir Academic Press; 2003. pp. 41–76.

79. Milek KB, Roberts HM. Integrated geoarchaeological methods for the determination of site activity areas: A study of a Viking Age house in Reykjavik, Iceland. J Archaeol Sci. 2013;40: 1845–1865. doi:10.1016/j.jas.2012.10.031

80. Scheschkewitz J, Freier T. Mittelalterliches Gerlingen und Spuren einer bandkeramischen Siedlung: Ausgrabungen im Träuble-Areal. Archäologische Ausgrabungen Baden-württemb. 2012; 244–248.

81. Zimmermann WH. Anmerkungen zur Geschichte des Stalles von der Urgeschichte bis zur Neuzeit am Beispiel von Rinderstall und Schweinekoben. Praehistorica. 2014;32: 329–358.

82. Carver M, Barrett J, Downes J, Hooper J, Sheridan A, Hunter F, et al. Pictish Byre-houses at Pitcarmick and their landscape: Investigations 1993-5. Proc Soc Antiq Scotl. 2012;142: 145–199.
