# Supplementary material for: High-resolution sediment analysis reveals Middle Bronze Age byre-houses at the site of Oppeano (Verona province, NE Italy)
Source: PLoS One. 2022 Aug 31;17(8):e0272561. doi: 10.1371/journal.pone.0272561 (PMC9432763; doi:10.1371/journal.pone.0272561)
Supplement: S2 Table — Asterisks mark the dates coming from structures E and F, studied here (see Figs 2 and 5). (DOCX) [file pone.0272561.s002.docx]

| **Sample no.** | **Sample Id** | **Structure** | **Stratigraphic Unit** | **Material** | **Lab ID** | **14C Age (yr BP)** | **Calibrated Age**  **(yr BC)** |
| --- | --- | --- | --- | --- | --- | --- | --- |
| 1 | OPP107 CARB 9 | A | US 393 | Charcoal (handpicked) | GrM 26663 | 3397 ± 29 | 1863BC-1854BC (1.0%) 1767BC-1614BC (94.4%) |
| **2*** | **OPP80 US308** | **E** | **US 308** | **Charcoal (handpicked)** | **GrM 27192** | **3476 ± 27** | **1884BC-1738BC (90.6%) 1712BC-1697BC (4.9%)** |
| 3 | OPP67  CARB 3 | L | US 407E | Charcoal (handpicked) | GrM 26656 | 3360 ± 35 | 1740BC-1710BC (11.6%) 1698BC-1536BC (83.8%) |
| **4*** | **OPPEANO BULK 2** | **F** | **US 561** | **Charcoal (handpicked)** | **RoAMS 1461.116** | **3301 ± 40** | **1686BC-1497BC (94.3%) 1473BC-1461BC (1.2%)** |
| **5*** | **OPP MON82 US619** | **E** | **US 618=619** | **Charcoal (handpicked)** | **RoAMS 1460.116** | **3360 ± 40** | **1744BC-1532BC (95.4%)** |
| 6 | OPP94  CARB 1 | C | US 699 | Charcoal (handpicked) | GrM 26661 | 3330 ± 30 | 1687BC-1517BC (95.4%) |
| 7 | OPP104 CARB 6 | G | US 674 | Charcoal (handpicked) | GrM 26662 | 3321 ± 29 | 1679BC-1654BC (4.7%) 1641BC-1510BC (90.8%) |
| 8 | OPP70  CARB 1 | L | US 407/366 | Charcoal (handpicked) | GrM 26658 | 3325 ± 30 | 1686BC-1651BC (7.8%) 1644BC-1511BC (87.7%) |
