# Supplementary material for: High-resolution sediment analysis reveals Middle Bronze Age byre-houses at the site of Oppeano (Verona province, NE Italy)
Source: PLoS One. 2022 Aug 31;17(8):e0272561. doi: 10.1371/journal.pone.0272561 (PMC9432763; doi:10.1371/journal.pone.0272561)
Supplement: S1 Appendix — For each thin section, the different scans (PPL and XPL), the subdivision in sub-units, and the interpreted version are available as overlapping layers that can be turned on and off using a PDF file reader. For a correct visualization, Adobe Acrobat is recommended. If one prefers a browser visualization, the plug-in Adobe PDF reader (free download) offers the same a correct visualization. Information on how to install and use the plug-in Adobe PDF reader can be find at this link: https://helpx.adobe.com/acrobat/using/display-pdf-in-browser.html (updated on May 4th, 2022). In Adobe PDF software and plug-in, the “layers” panel can be found and works as it follows: Choose View > Show/Hide > Navigation Panes > Layers.To hide a layer, click the eye icon. To show a hidden layer, click the empty box (a layer is visible when the eye icon is present, and hidden when the eye icon is absent).From the options menu, choose one of the following: List Layers For All Pages. Additional information can be found at this link: https://helpx.adobe.com/acrobat/using/pdf-layers.html (updated on May 4th, 2022). (PDF) [file pone.0272561.s003.pdf]

OPP73

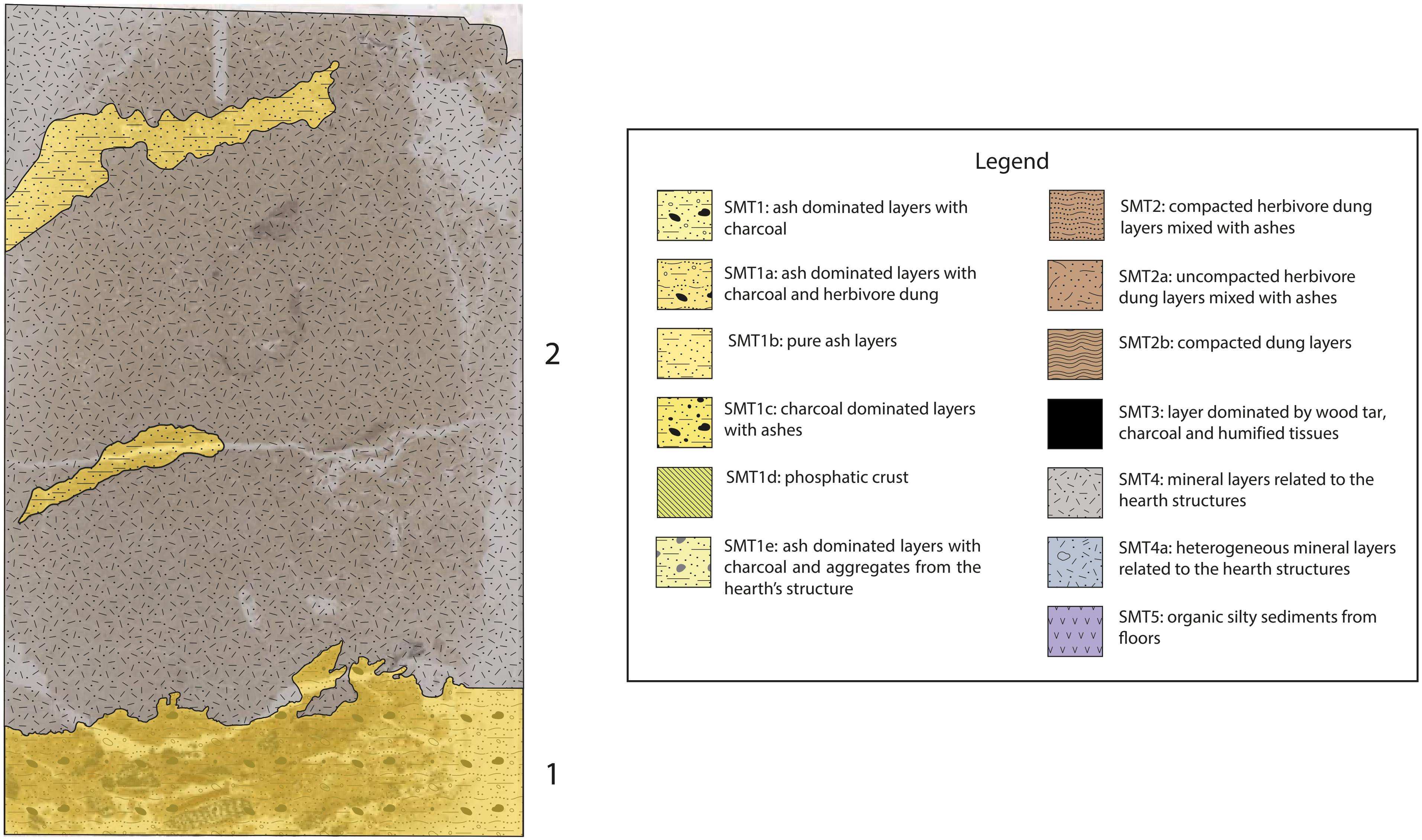
2
1
Legend
SMT1: ash dominated layers with charcoal
SMT1a: ash dominated layers with charcoal and herbivore dung
SMT1b: pure ash layers
SMT1c: charcoal dominated layers with ashes
SMT1d: phosphatic crust
SMT1e: ash dominated layers with charcoal and aggregates from the hearth's structure
SMT2: compacted herbivore dung layers mixed with ashes
SMT2a: uncompacted herbivore dung layers mixed with ashes
SMT2b: compacted dung layers
SMT3: layer dominated by wood tar, charcoal and humified tissues
SMT4: mineral layers related to the hearth structures
SMT4a: heterogeneous mineral layers related to the hearth structures
SMT5: organic silty sediments from floors

OPP 74

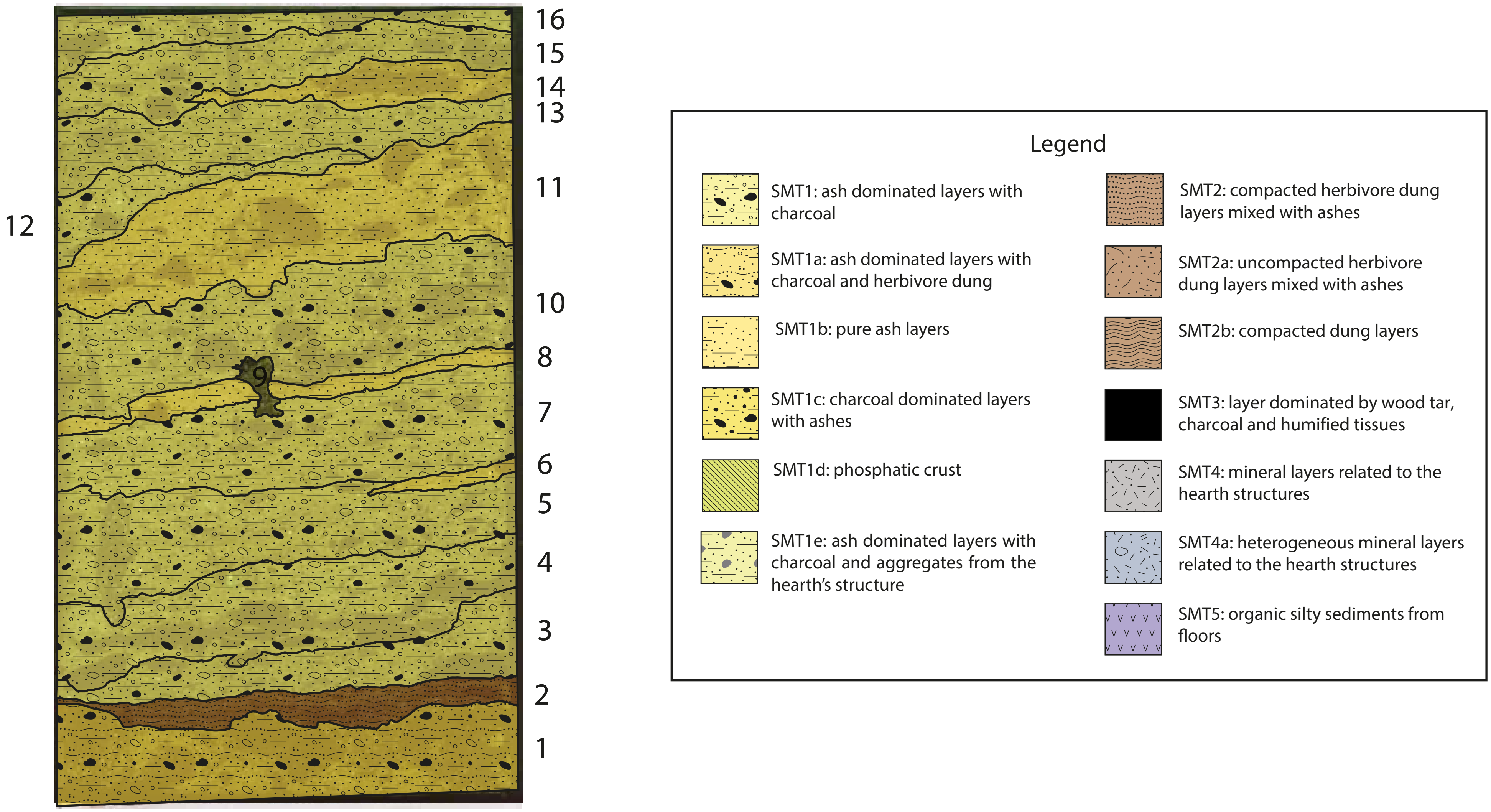
16
15
14
13
11
12
10
8
9
7
6
5
4
3
2
1
Legend
SMT1: ash dominated layers with charcoal
SMT1a: ash dominated layers with charcoal and herbivore dung
SMT1b: pure ash layers
SMT1c: charcoal dominated layers with ashes
SMT1d: phosphatic crust
SMT1e: ash dominated layers with charcoal and aggregates from the hearth's structure
SMT2: compacted herbivore dung layers mixed with ashes
SMT2a: uncompacted herbivore dung layers mixed with ashes
SMT2b: compacted dung layers
SMT3: layer dominated by wood tar, charcoal and humified tissues
SMT4: mineral layers related to the hearth structures
SMT4a: heterogeneous mineral layers related to the hearth structures
SMT5: organic silty sediments from floors

OPP 75

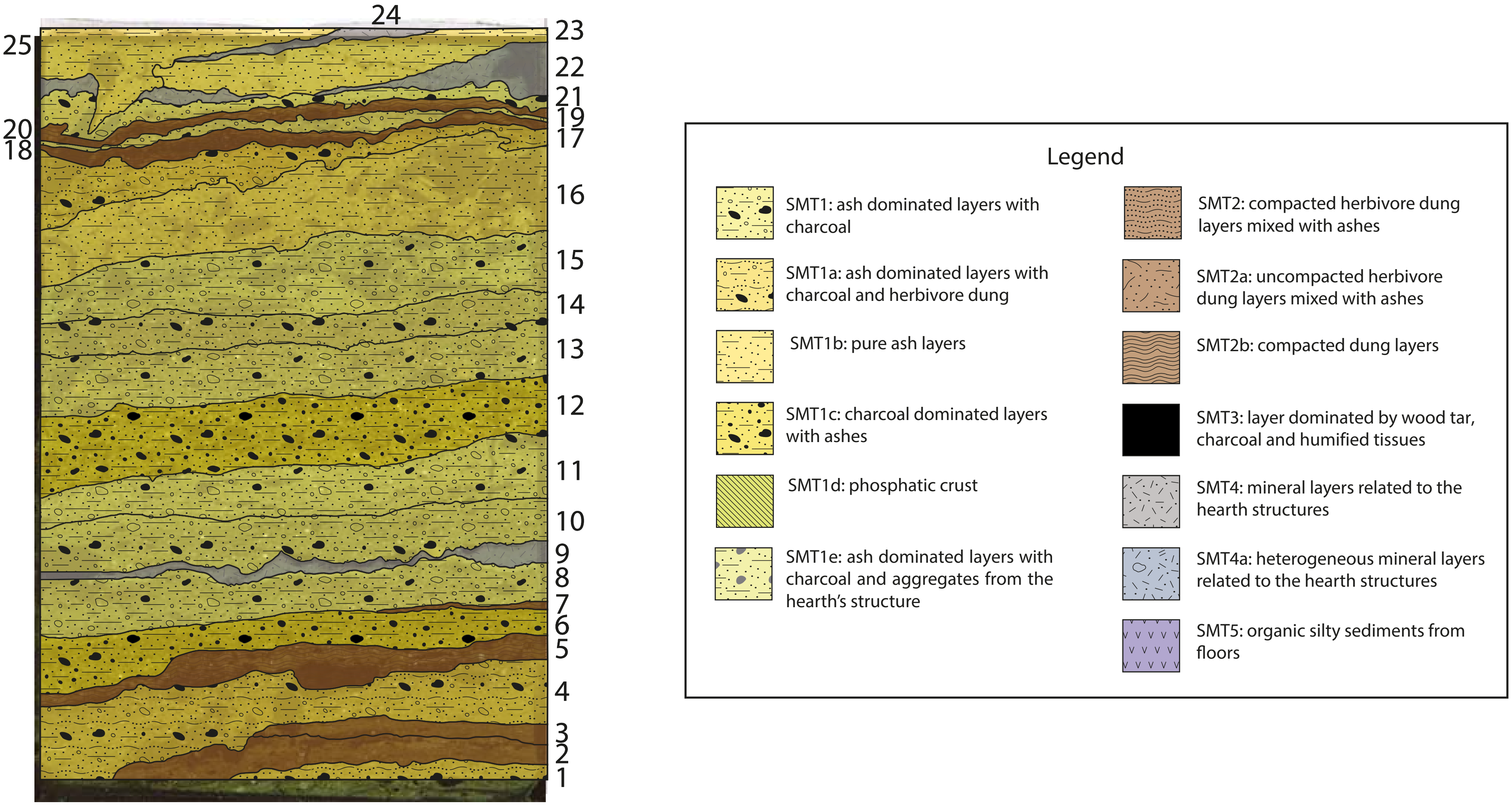

24
25
23
22
21
20
19
18
17
16
15
14
13
12
11
10
9
8
7
6
5
4
3
2
1
Legend
SMT1: ash dominated layers with charcoal
SMT1a: ash dominated layers with charcoal and herbivore dung
SMT1b: pure ash layers
SMT1c: charcoal dominated layers with ashes
SMT1d: phosphatic crust
SMT1e: ash dominated layers with charcoal and aggregates from the hearth's structure
SMT2: compacted herbivore dung layers mixed with ashes
SMT2a: uncompacted herbivore dung layers mixed with ashes
SMT2b: compacted dung layers
SMT3: layer dominated by wood tar, charcoal and humified tissues
SMT4: mineral layers related to the hearth structures
SMT4a: heterogeneous mineral layers related to the hearth structures
SMT5: organic silty sediments from floors

OPP 76

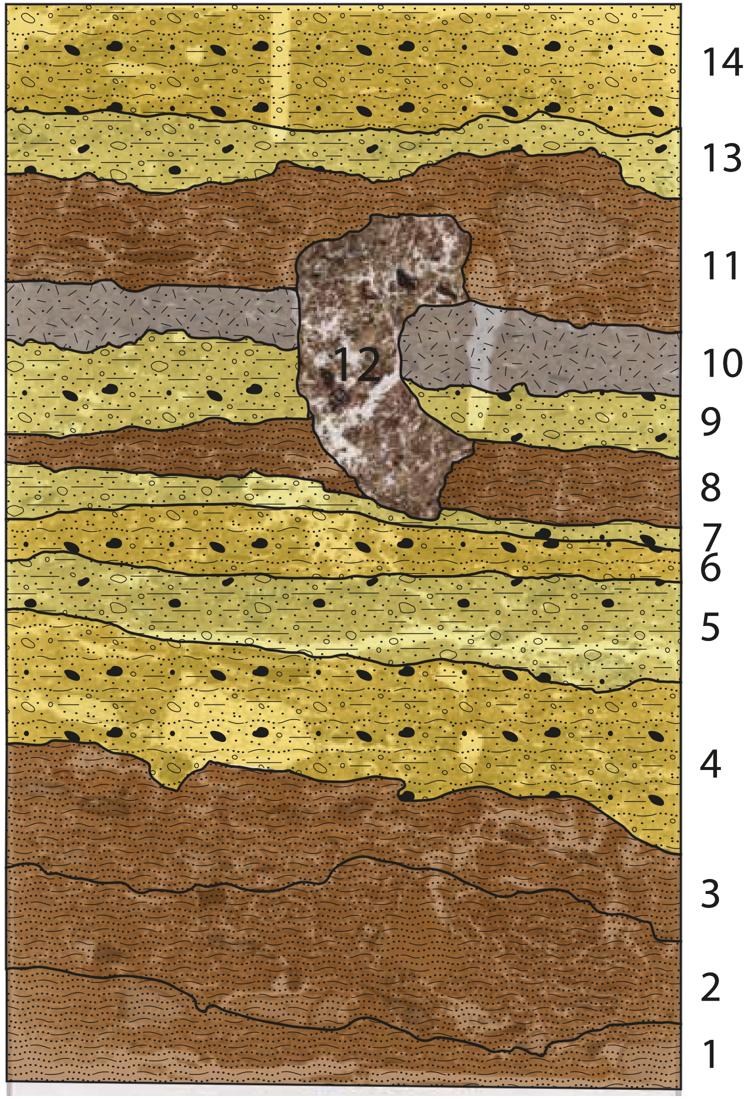

Legend

- SMT1: ash dominated layers with charcoal
- SMT1a: ash dominated layers with charcoal and herbivore dung
- SMT1b: pure ash layers
- SMT1c: charcoal dominated layers with ashes
- SMT1d: phosphatic crust
- SMT1e: ash dominated layers with charcoal and aggregates from the hearth's structure
- SMT2: compacted herbivore dung layers mixed with ashes
- SMT2a: uncompacted herbivore dung layers mixed with ashes
- SMT2b: compacted dung layers
- SMT3: layer dominated by wood tar, charcoal and humified tissues
- SMT4: mineral layers related to the hearth structures
- SMT4a: heterogeneous mineral layers related to the hearth structures
- SMT5: organic silty sediments from floors

OPP 77

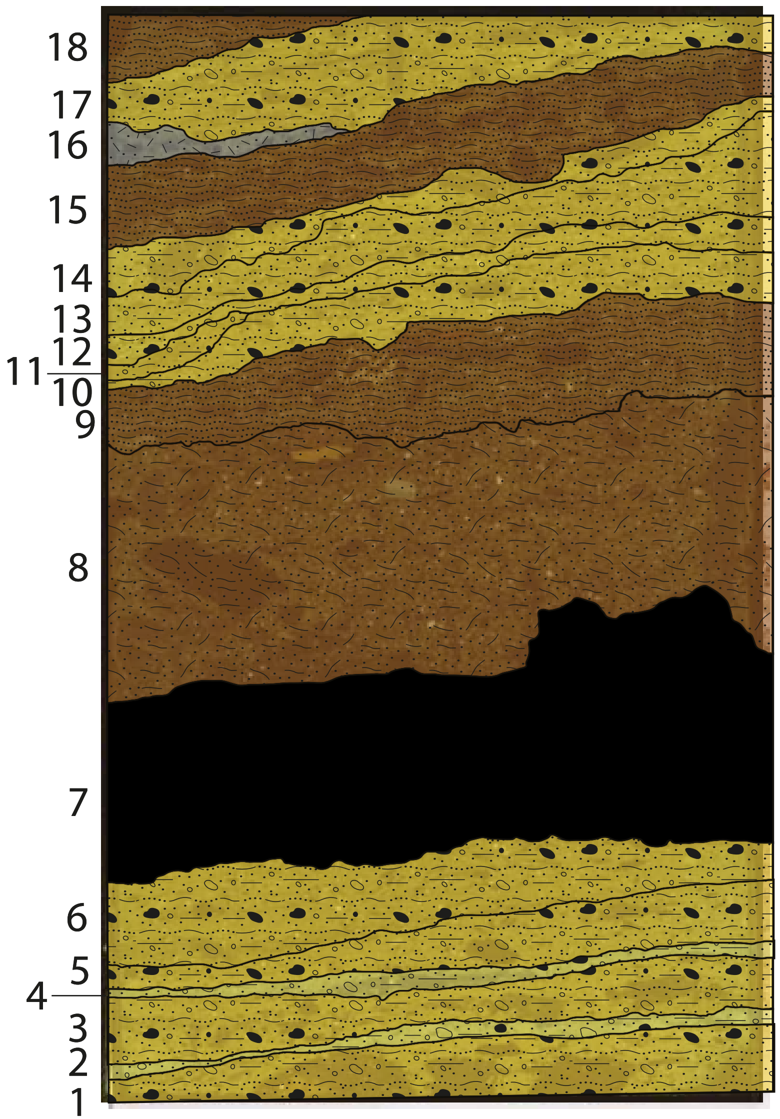

Legend

- SMT1: ash dominated layers with charcoal
- SMT1a: ash dominated layers with charcoals and herbivore dung
- SMT1b: pure ash layer
- SMT1c: charcoal dominated layers with ashes
- SMT1d: phosphatic crust
- SMT1e: ash dominated layers with charcoal and aggregates from the hearth's structure
- SMT2: compacted herbivore dung layers mixed with ashes
- SMT2a: uncompacted herbivore dung layers mixed with ashes
- SMT2b: compacted dung layers
- SMT3: layer dominated by wood tar, charcoal and humified tissues
- SMT4: mineral layers related to the hearth structures
- SMT4a: heterogeneous mineral layers related to the hearth structures
- SMT5: organic silty sediments from floors

OPP 78

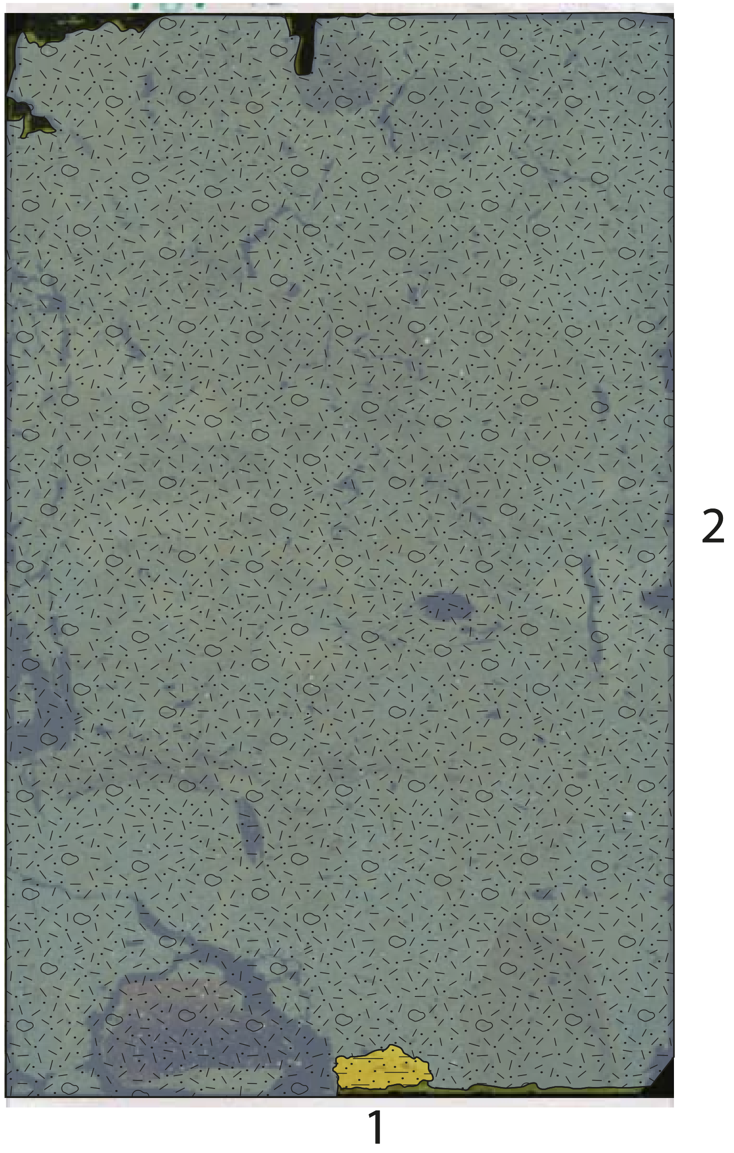

Legend

SMT1: ash dominated layers with charcoal

SMT1a: ash dominated layers with charcoal and herbivore dung

SMT1b: pure ash layers

SMT1c: charcoal dominated layers with ashes

SMT1d: phosphatic crust

SMT1e: ash dominated layers with charcoal and aggregates from the hearth's structure

SMT2: compacted herbivore dung layers mixed with ashes

SMT2a: uncompacted herbivore dung layers mixed with ashes

SMT2b: compacted dung layers

SMT3: layer dominated by wood tar, charcoal and humified tissues

SMT4: mineral layers related to the hearth structures

SMT4a: heterogeneous mineral layers related to the hearth structures

SMT5: organic silty sediments from floors

OPP 79

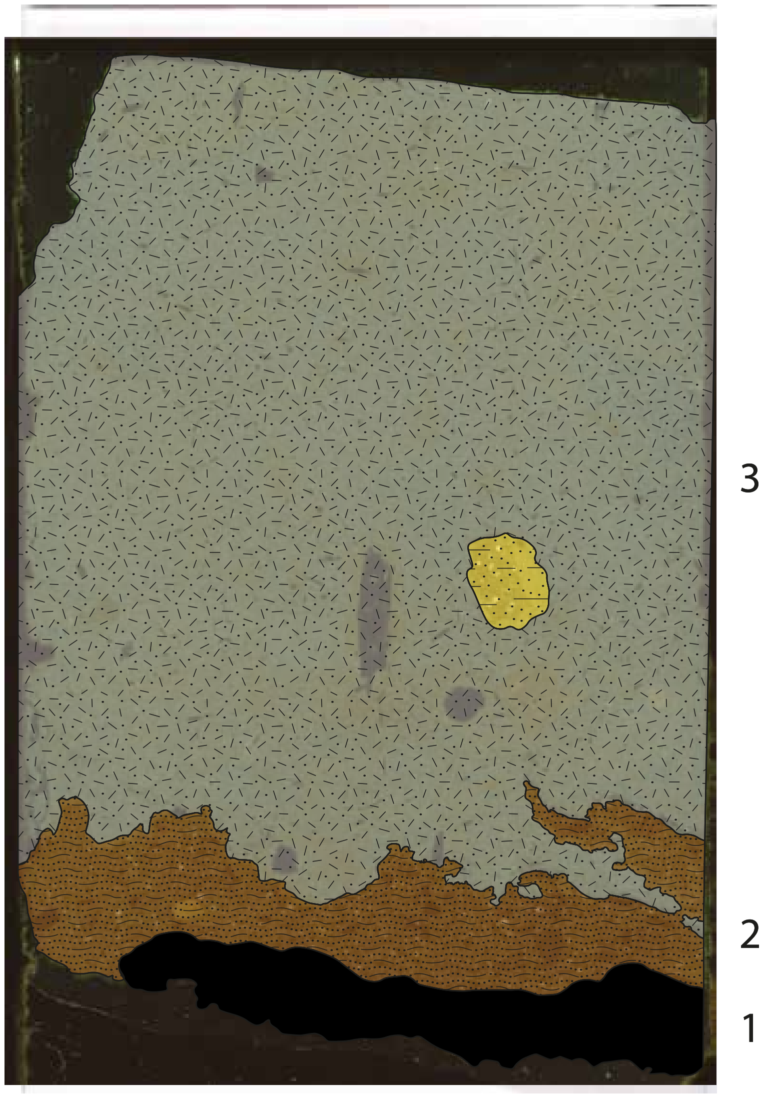

Legend

- SMT1: ash dominated layers with charcoal
- SMT1a: ash dominated layers with charcoal and herbivore dung
- SMT1b: pure ash layers
- SMT1c: charcoal dominated layers with ashes
- SMT1d: phosphatic crust
- SMT1e: ash dominated layers with charcoal and aggregates from the hearth's structure
- SMT2: compacted herbivore dung layers mixed with ashes
- SMT2a: uncompacted herbivore dung layers mixed with ashes
- SMT2b: compacted dung layers
- SMT3: layer dominated by wood tar, charcoal and humified tissues
- SMT4: mineral layers related to the hearth structures
- SMT4a: heterogeneous mineral layers related to the hearth structures
- SMT5: organic silty sediments from floors

OPP 80-1

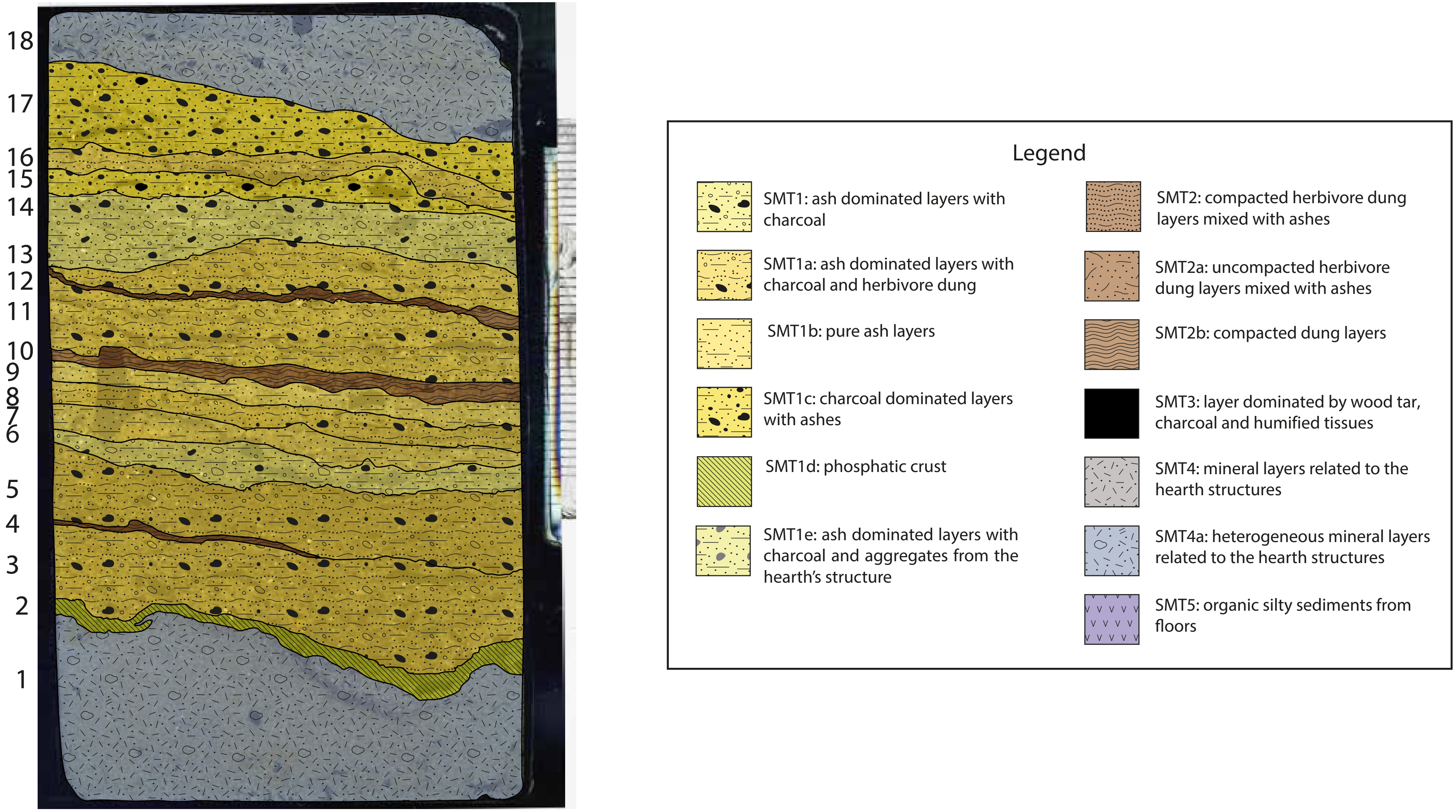
18
17
16
15
14
13
12
11
10
9
8
7
6
5
4
3
2
1
Legend
SMT1: ash dominated layers with charcoal
SMT1a: ash dominated layers with charcoal and herbivore dung
SMT1b: pure ash layers
SMT1c: charcoal dominated layers with ashes
SMT1d: phosphatic crust
SMT1e: ash dominated layers with charcoal and aggregates from the hearth's structure
SMT2: compacted herbivore dung layers mixed with ashes
SMT2a: uncompacted herbivore dung layers mixed with ashes
SMT2b: compacted dung layers
SMT3: layer dominated by wood tar, charcoal and humified tissues
SMT4: mineral layers related to the hearth structures
SMT4a: heterogeneous mineral layers related to the hearth structures
SMT5: organic silty sediments from floors

OPP 80-2

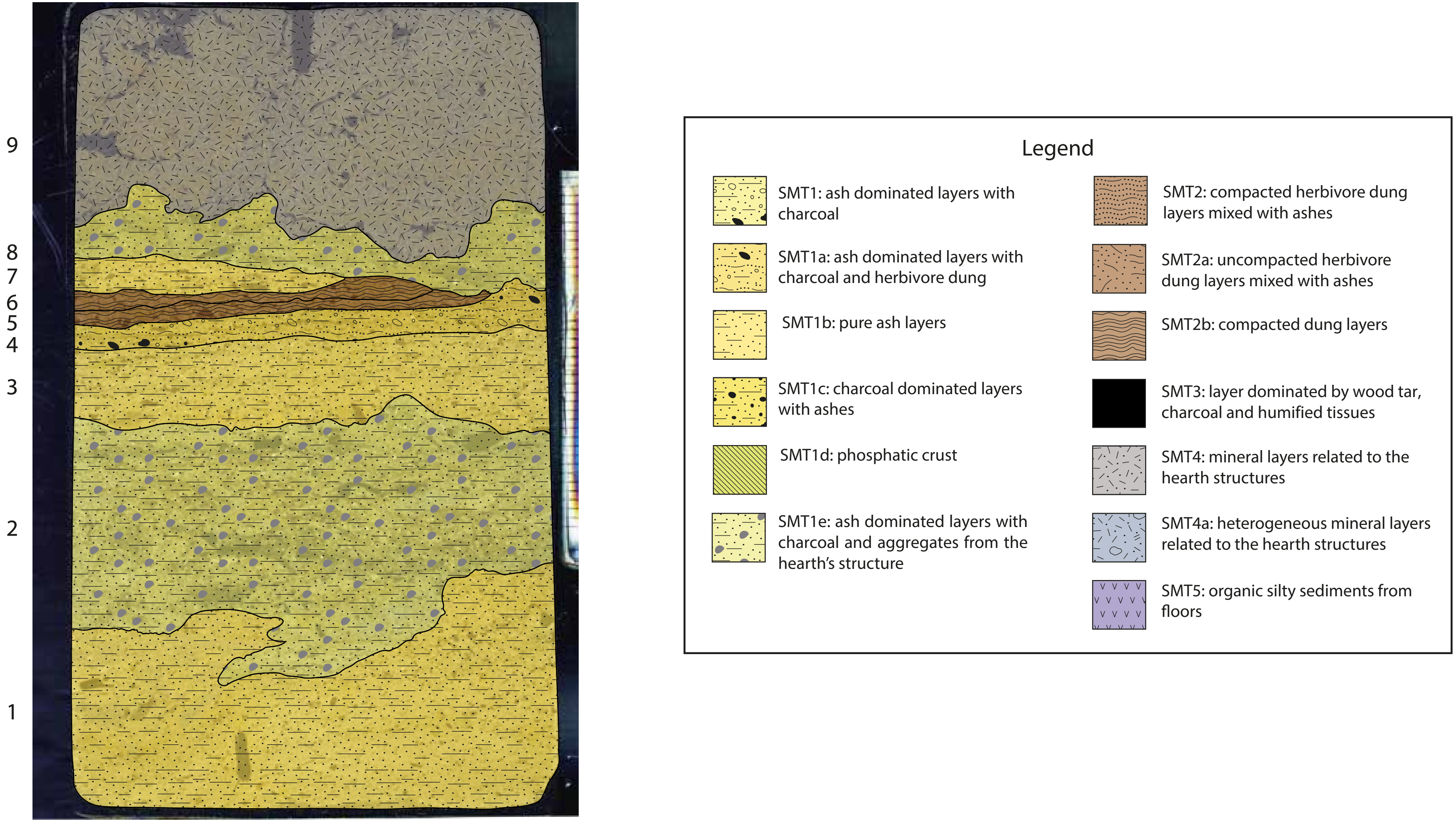
9
8
7
6
5
4
3
2
1
Legend
SMT1: ash dominated layers with charcoal
SMT1a: ash dominated layers with charcoal and herbivore dung
SMT1b: pure ash layers
SMT1c: charcoal dominated layers with ashes
SMT1d: phosphatic crust
SMT1e: ash dominated layers with charcoal and aggregates from the hearth's structure
SMT2: compacted herbivore dung layers mixed with ashes
SMT2a: uncompacted herbivore dung layers mixed with ashes
SMT2b: compacted dung layers
SMT3: layer dominated by wood tar, charcoal and humified tissues
SMT4: mineral layers related to the hearth structures
SMT4a: heterogeneous mineral layers related to the hearth structures
SMT5: organic silty sediments from floors

OPP 81-1

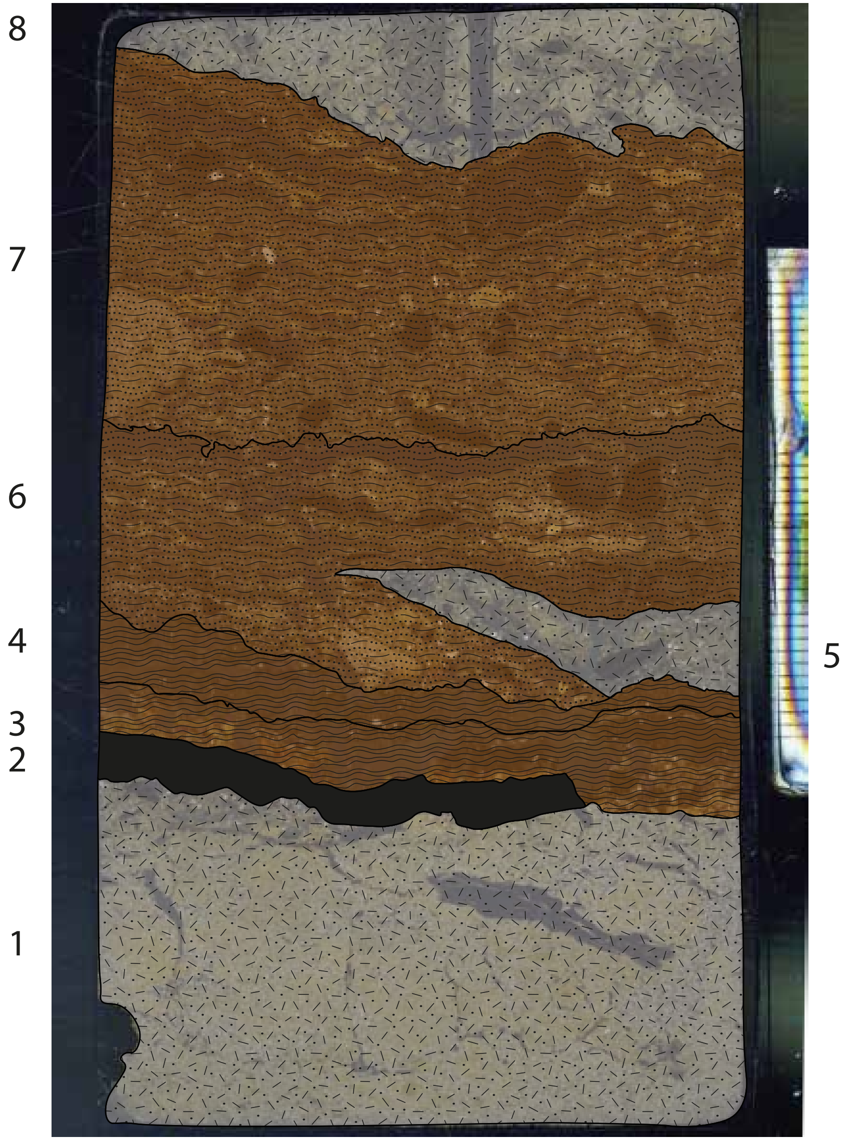

Legend

- SMT1: ash dominated layers with charcoal
- SMT1a: ash dominated layers with charcoal and herbivore dung
- SMT1b: pure ash layers
- SMT1c: charcoal dominated layers with ashes
- SMT1d: phosphatic crust
- SMT1e: ash dominated layers with charcoal and aggregates from the hearth's structure
- SMT2: compacted herbivore dung layers mixed with ashes
- SMT2a: uncompacted herbivore dung layers mixed with ashes
- SMT2b: compacted dung layers
- SMT3: layer dominated by wood tar, charcoal and humified tissues
- SMT4: mineral layers related to the hearth structures
- SMT4a: heterogeneous mineral layers related to the hearth structures
- SMT5: organic silty sediments from floors

OPP 81-2

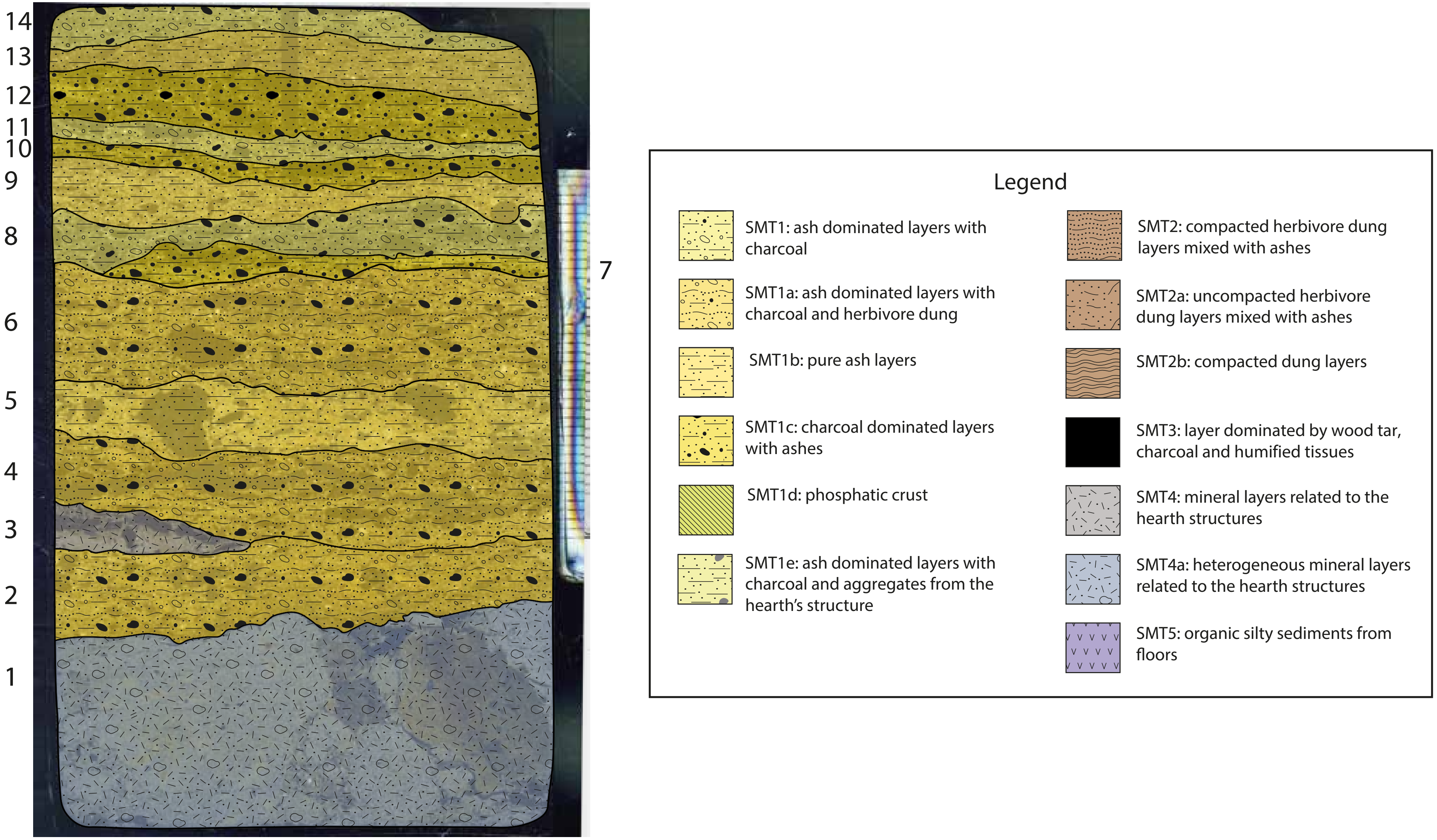

14
13
12
11
10
9
8
7
6
5
4
3
2
1
Legend
SMT1: ash dominated layers with charcoal
SMT1a: ash dominated layers with charcoal and herbivore dung
SMT1b: pure ash layers
SMT1c: charcoal dominated layers with ashes
SMT1d: phosphatic crust
SMT1e: ash dominated layers with charcoal and aggregates from the hearth's structure
SMT2: compacted herbivore dung layers mixed with ashes
SMT2a: uncompacted herbivore dung layers mixed with ashes
SMT2b: compacted dung layers
SMT3: layer dominated by wood tar, charcoal and humified tissues
SMT4: mineral layers related to the hearth structures
SMT4a: heterogeneous mineral layers related to the hearth structures
SMT5: organic silty sediments from floors

OPP 81-3

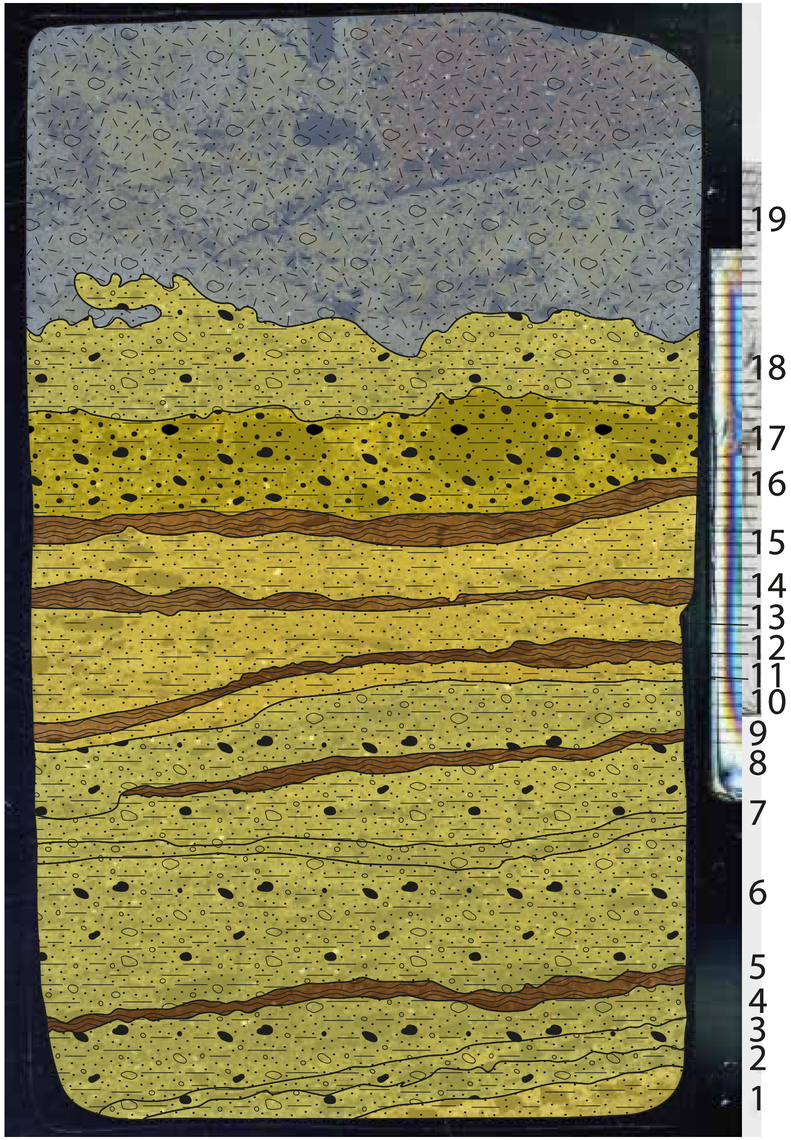

Legend

- SMT1: ash dominated layers with charcoal
- SMT1a: ash dominated layers with charcoal and herbivore dung
- SMT1b: pure ash layers
- SMT1c: charcoal dominated layers with ashes
- SMT1d: phosphatic crust
- SMT1e: ash dominated layers with charcoal and aggregates from the hearth's structure
- SMT2: compacted herbivore dung layers mixed with ashes
- SMT2a: uncompacted herbivore dung layers mixed with ashes
- SMT2b: compacted dung layers
- SMT3: layer dominated by wood tar, charcoal and humified tissues
- SMT4: mineral layers related to the hearth structures
- SMT4a: heterogeneous mineral layers related to the hearth structures
- SMT5: organic silty sediments from floors

OPP 82-1

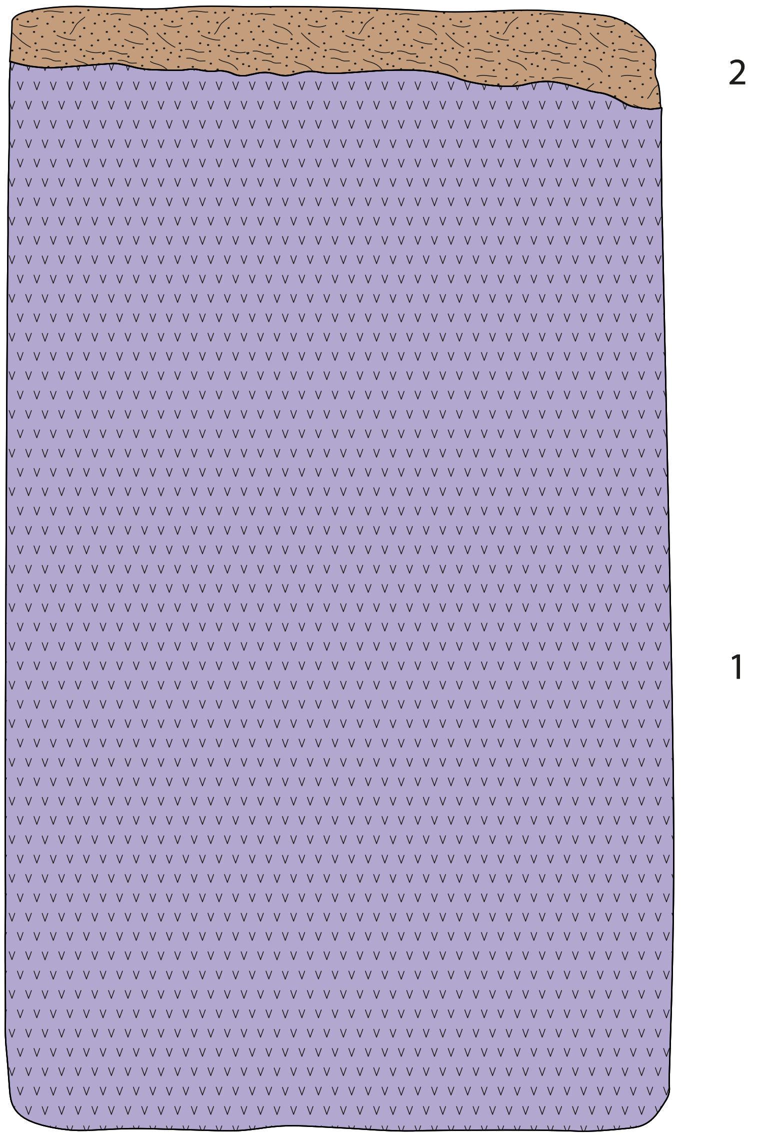

Legend

- SMT1: ash dominated layers with charcoal
- SMT1a: ash dominated layers with charcoal and herbivore dung
- SMT1b: pure ash layers
- SMT1c: charcoal dominated layers with ashes
- SMT1d: phosphatic crust
- SMT1e: ash dominated layers with charcoal and aggregates from the hearth's structure
- SMT2: compacted herbivore dung layers mixed with ashes
- SMT2a: uncompacted herbivore dung layers mixed with ashes
- SMT2b: compacted dung layers
- SMT3: layer dominated by wood tar, charcoal and humified tissues
- SMT4: mineral layers related to the hearth structures
- SMT4a: heterogeneous mineral layers related to the hearth structures
- SMT5: organic silty sediments from floors

OPP 82-2

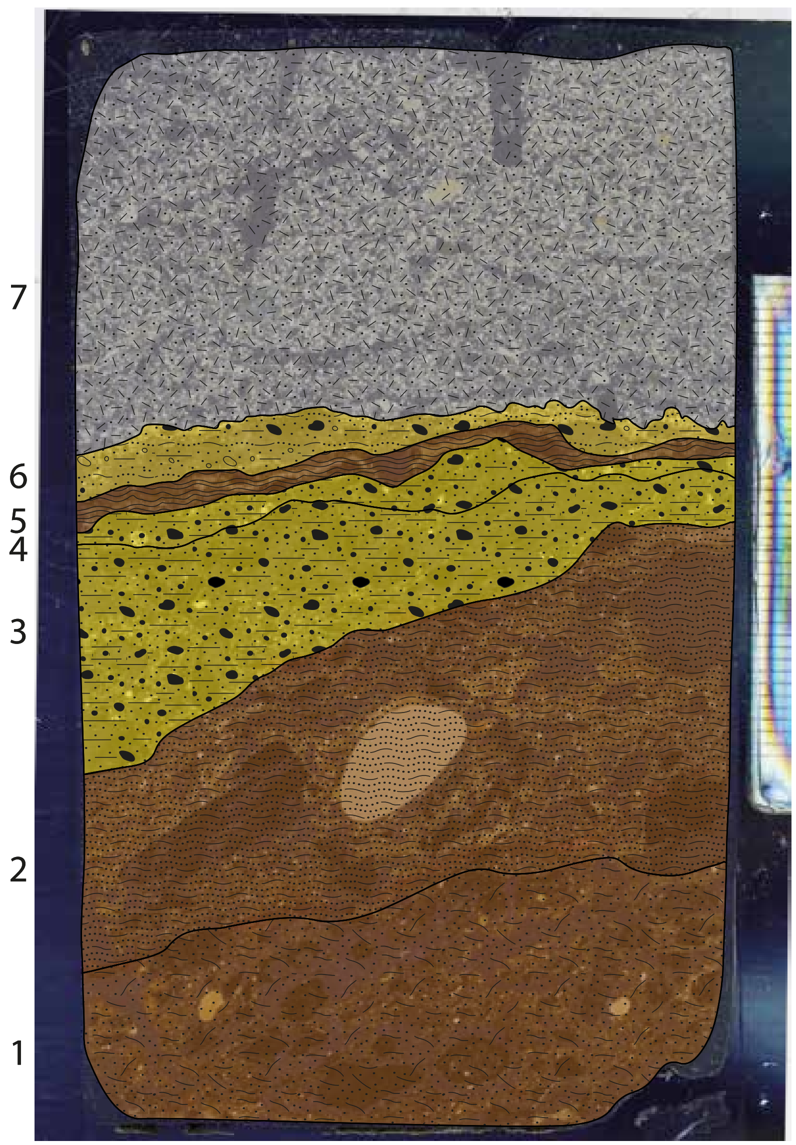

Legend

- SMT1: ash dominated layers with charcoal
- SMT1a: ash dominated layers with charcoal and herbivore dung
- SMT1b: pure ash layers
- SMT1c: charcoal dominated layers with ashes
- SMT1d: phosphatic crust
- SMT1e: ash dominated layers with charcoal and aggregates from the hearth's structure
- SMT2: compacted herbivore dung layers mixed with ashes
- SMT2a: uncompacted herbivore dung layers mixed with ashes
- SMT2b: compacted dung layers
- SMT3: layer dominated by wood tar, charcoal and humified tissues
- SMT4: mineral layers related to the hearth structures
- SMT4a: heterogeneous mineral layers related to the hearth structures
- SMT5: organic silty sediments from floors

OPP 82-3

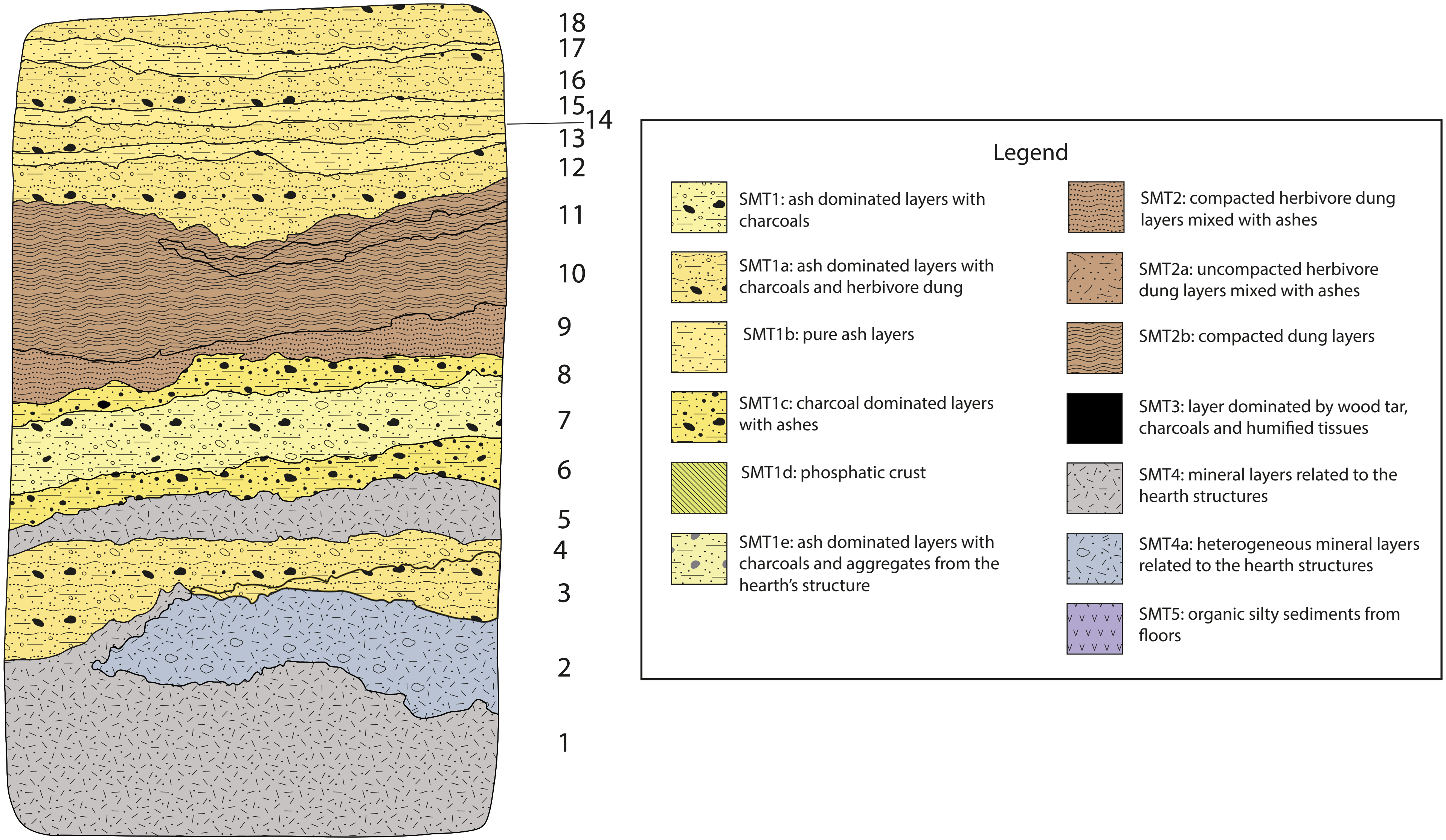
18
17
16
15
14
13
12
11
10
9
8
7
6
5
4
3
2
1
Legend
SMT1: ash dominated layers with charcoals
SMT1a: ash dominated layers with charcoals and herbivore dung
SMT1b: pure ash layers
SMT1c: charcoal dominated layers with ashes
SMT1d: phosphatic crust
SMT1e: ash dominated layers with charcoals and aggregates from the hearth's structure
SMT2: compacted herbivore dung layers mixed with ashes
SMT2a: uncompacted herbivore dung layers mixed with ashes
SMT2b: compacted dung layers
SMT3: layer dominated by wood tar, charcoals and humified tissues
SMT4: mineral layers related to the hearth structures
SMT4a: heterogeneous mineral layers related to the hearth structures
SMT5: organic silty sediments from floors
